# Investigating the Impact of Environment and Data Aggregation by Walking Bout Duration on Parkinson’s Disease Classification Using Machine Learning

**DOI:** 10.3389/fnagi.2022.808518

**Published:** 2022-03-22

**Authors:** Rana Zia Ur Rehman, Yu Guan, Jian Qing Shi, Lisa Alcock, Alison J. Yarnall, Lynn Rochester, Silvia Del Din

**Affiliations:** ^1^Translational and Clinical Research Institute, Newcastle University, Newcastle upon Tyne, United Kingdom; ^2^School of Computing, Newcastle University, Newcastle upon Tyne, United Kingdom; ^3^School of Mathematics, Statistics and Physics, Newcastle University, Newcastle upon Tyne, United Kingdom; ^4^Department of Statistics and Data Science, Southern University of Science and Technology, Shenzhen, China; ^5^The Newcastle upon Tyne Hospitals NHS Foundation Trust, Newcastle upon Tyne, United Kingdom

**Keywords:** Parkinson’s disease, gait, real-world, accelerometer, machine learning, laboratory, gait aggregation, wearables

## Abstract

Parkinson’s disease (PD) is a common neurodegenerative disease. PD misdiagnosis can occur in early stages. Gait impairment in PD is typical and is linked with an increased fall risk and poorer quality of life. Applying machine learning (ML) models to real-world gait has the potential to be more sensitive to classify PD compared to laboratory data. Real-world gait yields multiple walking bouts (WBs), and selecting the optimal method to aggregate the data (e.g., different WB durations) is essential as this may influence classification performance. The objective of this study was to investigate the impact of environment (laboratory vs. real world) and data aggregation on ML performance for optimizing sensitivity of PD classification. Gait assessment was performed on 47 people with PD (age: 68 ± 9 years) and 52 controls [Healthy controls (HCs), age: 70 ± 7 years]. In the laboratory, participants walked at their normal pace for 2 min, while in the real world, participants were assessed over 7 days. In both environments, 14 gait characteristics were evaluated from one tri-axial accelerometer attached to the lower back. The ability of individual gait characteristics to differentiate PD from HC was evaluated using the Area Under the Curve (AUC). ML models (i.e., support vector machine, random forest, and ensemble models) applied to real-world gait showed better classification performance compared to laboratory data. Real-world gait characteristics aggregated over longer WBs (WB 30–60 s, WB > 60 s, WB > 120 s) resulted in superior discriminative performance (PD vs. HC) compared to laboratory gait characteristics (0.51 ≤ AUC ≤ 0.77). Real-world gait speed showed the highest AUC of 0.77. Overall, random forest trained on 14 gait characteristics aggregated over WBs > 60 s gave better performance (F1 score = 77.20 ± 5.51%) as compared to laboratory results (F1 Score = 68.75 ± 12.80%). Findings from this study suggest that the choice of environment and data aggregation are important to achieve maximum discrimination performance and have direct impact on ML performance for PD classification. This study highlights the importance of a harmonized approach to data analysis in order to drive future implementation and clinical use.

**Clinical Trial Registration:**

[09/H0906/82].

## Introduction

Parkinson’s disease (PD) is the second most common neurodegenerative disease after Alzheimer’s disease (Nutt and Wooten, 2005; Feigin et al., 2019). PD prevalence has doubled over the past 25 years and now affects approximately 10 million people worldwide (Dorsey et al., 2018). Due to the progressive nature of PD (Dorsey et al., 2013; Emamzadeh and Surguchov, 2018), both motor (Hobert et al., 2019) and non-motor (Przedborski et al., 2003; Jankovic, 2008) symptoms have a significant impact on quality of life and increased burden on healthcare costs (von Campenhausen et al., 2011). Currently, diagnostic criteria for PD are based on motor features assessed with clinical scales (Jankovic, 2008; Postuma et al., 2015). However, the diagnostic accuracy of PD in a clinical setting is only 74% if performed by a non-expert and 80% by a movement disorder specialist (Rizzo et al., 2016). Given the relatively low rates of accurate diagnosis, particularly in the early stages, there is a need for additional diagnostic aids (Mancini et al., 2011). The application of gait analysis may be a promising addition to the diagnostic toolkit (Buckley et al., 2019; Viceconti et al., 2020).

Previous work has shown that an objective gait assessment obtained in lab settings and the clinic can be used to classify PD using machine learning (ML) approaches (Rehman et al., 2019a,b, 2020a,b). However, assessing gait in both the lab and the clinic has some key limitations. The patient is required to attend specialist facilities, and assessments often do not represent the range of challenges associated with habitual walking (Orendurff et al., 2008). Moreover, individuals tend to perform better (walk faster) during performance tests which reflects walking capacity (“can do”) (Del Din et al., 2016a) compared to everyday life which captures the functional performance (“actually do”) of the participant (Hillel et al., 2019; Maetzler et al., 2020; Shah et al., 2020c; Warmerdam et al., 2020; Atrsaei et al., 2021). The real world may therefore provide a more sensitive and pragmatic context to identify and classify PD (Shah et al., 2020c). Increasing interest in the use of inertial measurement units (IMUs) to monitor gait in people with PD in the lab is evident (González et al., 2010; McCamley et al., 2012; Zijlstra and Zijlstra, 2013; Godfrey et al., 2015; Del Din et al., 2016c), as is monitoring gait continuously in the real world over multiple consecutive days (De Bruin et al., 2007; Weiss et al., 2013, 2014; Godfrey et al., 2014). However, several methodological challenges remain for a better understanding and analysis of real-world gait data. These include extraction of relevant gait characteristics and appropriate use of data aggregation for analysis, e.g., averaging gait characteristics using various WB durations.

Spatiotemporal gait characteristics [from the gait domains of pace, rhythm, variability, asymmetry, and postural control (Lord et al., 2013)] from lab and real-world data are significantly different in people with PD compared to healthy controls (HCs) (Maetzler et al., 2013; Del Din et al., 2016a). However, methods for analysis of data obtained in real-world settings rely on selecting the protocol for gait assessment (e.g., environment and duration) and data aggregation by walking bout (WB) duration (e.g., aggregating all WB’s or selecting an optimal bout duration) (Del Din et al., 2016a,b, 2019; Shah et al., 2020a; Warmerdam et al., 2020). All these options impact on the quantification of spatiotemporal gait characteristics and subsequent results (Del Din et al., 2016b).

Real-world gait consists of a variety of WBs of different durations, the majority of which are short (<10 s, approximately 50%) with only 3% over 60 s for both PD and HC (Del Din et al., 2016a). In contrast, lab-based gait assessments are based on standardized walking distances such as 4 or 10 m (Del Din et al., 2016a,c; Van Ancum et al., 2019) or duration (e.g., 2 min) (Rehman et al., 2019a,b; Del Din et al., 2020). Comparison of data obtained in the lab and in the real-world is therefore challenging.

In previous work, ML classifiers have been trained on data from lab-based gait assessments (Rehman et al., 2019a,b, 2020a,b). The impact of environment (lab vs. real-world) and data aggregation by WB duration on PD classification has not been thoroughly explored. Different WB durations also influence the distribution of gait characteristics. Therefore, ML models need to be able to account for multiple distributions (due to inclusion of a variety of short and long WBs) of real-world gait characteristics. To the best of the authors’ knowledge, the impact of WB durations on the classification of PD using machine learning approaches has not yet been investigated.

The aims of this study are therefore to: (i) investigate the impact of environment (gait assessment in lab vs. real world) and (ii) data aggregation by WB duration on gait characteristics and performance of ML models to accurately classify PD. Based on current available univariate analyses (Del Din et al., 2016a,^2019^; Shah et al., 2020c), we hypothesized that: (i) real-world gait would be more sensitive for performing the ML based classification of PD compared to lab gait assessment; (ii) associations between lab-based and real-world gait would vary depending on WB duration; and (iii) ML model performance would be influenced by WB duration.

## Materials and Methods

### Participants

In this cross-sectional analysis, 52 HCs and 47 people with PD were included from the 18 month time point of the “Incidence of Cognitive Impairment in Cohorts with Longitudinal Evaluation–GAIT” (ICICLE-GAIT) study (Khoo et al., 2013; Yarnall et al., 2014). ICICLE-GAIT is a study nested within ICICLE-PD which recruited participants between June 2009 and December 2011 (Khoo et al., 2013). PD participants were recruited from local movement disorders clinics (Khoo et al., 2013) and had a diagnosis of idiopathic PD according to the United Kingdom Brain Bank Criteria (Khoo et al., 2013; Yarnall et al., 2014). PD participants who have Parkinsonism disorders or an atypical form of Parkinson’s disease, with poor knowledge of working English language, or with cognitive impairment (Mini-Mental State Examination score < 24) were excluded from the study. The HC participants were recruited from the local community and included provided that they were able to walk independently and were without significant motor, mood, or cognitive impairment. ICICLE-GAIT received ethical approval from the Newcastle and North Tyneside Research Ethics Committee (REC No. 09/H0906/82). Study procedures were conducted according to the Declaration of Helsinki and all participants gave written informed consent prior to participating.

### Demographics and Clinical Characteristics

Participant demographics such as sex, age, mass, height, and BMI were recorded. The Montreal Cognitive Assessment (MoCA) was used to measure global cognition (Nasreddine et al., 2005). Balance confidence was assessed using the Activities Specific Balance Confidence scale (ABC) (Powell and Myers, 1995). PD motor severity was assessed with Part III of the Movement Disorder Society Unified Parkinson’s Disease Rating Scale (MDS-UPDRS III) (Goetz et al., 2008) and disease stages were also recorded according to Hoehn and Yahr (1998). The levodopa equivalent daily dose (LEDD; mg/day) was also calculated (Tomlinson et al., 2010; Lawson et al., 2016).

### Gait Assessment

#### Equipment and Protocol

**FIGURE 1:**
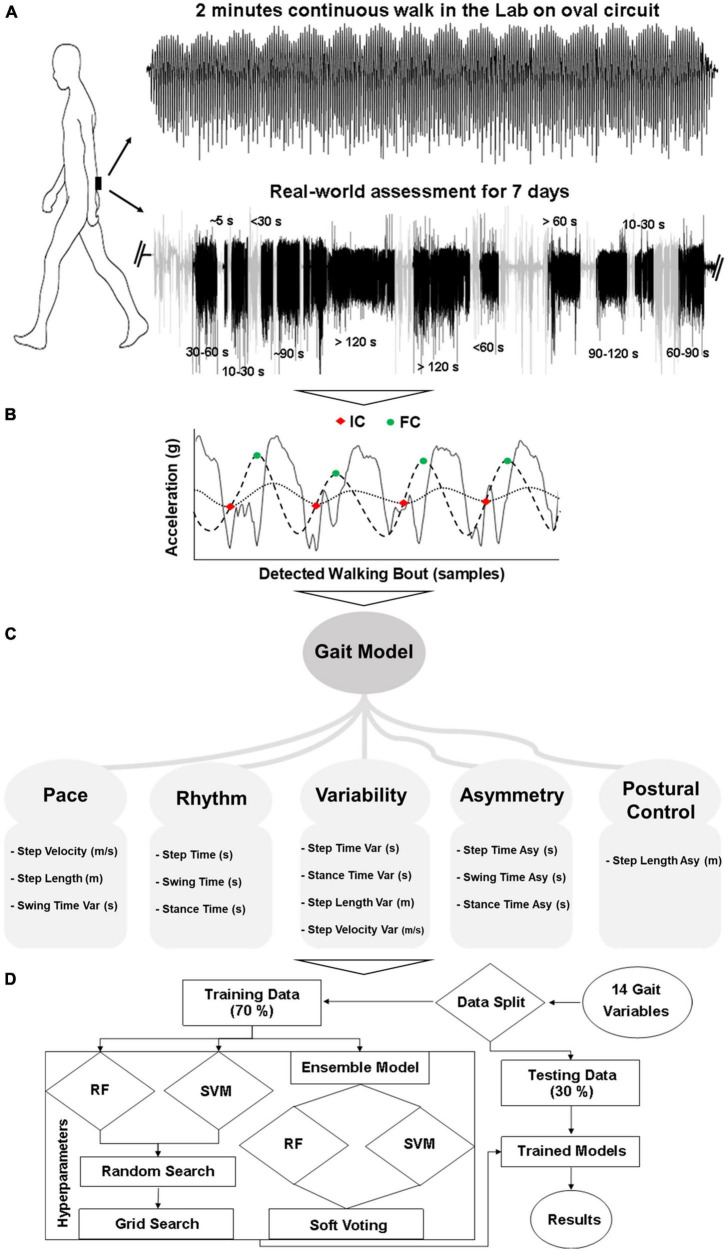
Overall workflow from gait assessment to classification: **(A)** Gait assessment protocol, **(B)** WB detection and gait characterization, **(C)** 14 gait characteristics (Var, variability; Asy, asymmetry), **(D)** Classification modeling.

Gait assessment was performed in the lab and real-world using a tri-axial (x: vertical, y: mediolateral, z: anteroposterior) accelerometer (Axivity, AX3, sample frequency: 100 Hz, range: ±8 g) on the lower back, as shown in Figure 1A. In the lab, a 2 min continuous walk around an oval circuit was performed (Rehman et al., 2019b). PD participants’ gait was assessed while optimally medicated (approximately one hour after medication intake). In the real-world, gait was monitored continuously for 7 days (Del Din et al., 2016a,b, 2019). This took place following the lab assessment. Participants were instructed to perform their usual activities. Further details can be found in previous work (Godfrey et al., 2014; Del Din et al., 2016a,^2019^).

#### Data Processing and Extraction of Gait Characteristics–Lab

Data from the accelerometer was downloaded to a computer for offline processing in MATLAB (R2019a). The vertical component of the transformed acceleration signal was filtered first to 20 Hz with a 4th order Butterworth filter (Moe-Nilssen, 1998; Zijlstra and Hof, 2003; McCamley et al., 2012). To detect the events within each gait cycle (Figure 1B), the initial contact (IC, heel strike) and final contact (FC, toe-off) points were identified with the help of a Gaussian continuous wavelet transform. Additional temporal gait characteristics (step time, swing time, and stance time) were quantified based on IC and FC (McCamley et al., 2012; Godfrey et al., 2015; Del Din et al., 2016c). For the evaluation of spatial characteristics (step length) the inverted pendulum model was utilized (Zijlstra and Zijlstra, 2013) and step velocity was calculated as the ratio of step length and step time (Del Din et al., 2016c). Variability was calculated as the standard deviation from all steps and asymmetry as the absolute difference of alternative steps (left and right) (Del Din et al., 2016c). The detailed method for the evaluation of spatiotemporal gait characteristics is described in previous work (Lord et al., 2013; Godfrey et al., 2015; Del Din et al., 2016c).

Fourteen spatiotemporal gait characteristics (Figure 1C) were extracted based on ICs and FCs and mapped onto five domains: pace (step velocity, step length, swing time variability), rhythm (step time, swing time, stance time), variability (step velocity variability, step length variability, step time variability, swing time variability, stance time variability), asymmetry (step time asymmetry, swing time asymmetry, stance time asymmetry), and postural control (step length asymmetry) (Lord et al., 2013; Godfrey et al., 2015; Del Din et al., 2016c).

#### Data Processing, Extraction of Gait Characteristics, and Data Aggregation–Real-World

For the real-world gait assessment, data was downloaded to a computer for offline processing in MATLAB (R2019a). Accelerometry data was segmented into each calendar day and WBs were detected based on the magnitude and standard deviation of the acceleration signal (Del Din et al., 2016d; Hickey et al., 2016). A WB was defined as the continuous length of time spent during walking (Godfrey et al., 2014), with at least three steps (Del Din et al., 2016a,^2019^). No resting period thresholds between consecutive WBs were set so that each WB was individually considered (and not merged to other WBs) (Barry et al., 2015). Gait characteristics were firstly evaluated for each WB by combining all steps within a WB. Then, all WBs were combined for each day to provide a daily average. Finally, each day was combined to provide a 7 day average for each gait characteristic (Del Din et al., 2016a,^2019^). The same fourteen gait characteristics were extracted from the real-world (Lord et al., 2013; Godfrey et al., 2015; Del Din et al., 2016c) for comparison with lab-based gait (Figure 1C).

To investigate the impact of real-world data aggregation by WB duration on gait characteristics and ML models, a comprehensive approach was adopted. WB of various durations (seconds) were considered and aggregated over the 7 days (Figure 1A). In total, fourteen WB durations were chosen, and the average of all WBs was used to describe each gait characteristic. The six most optimal and distinct WB durations without having redundant information by combining the incremental WBs (i.e., WBs ≤ 10 s, 10 < WBs ≤ 30 s, 30 < WBs ≤ 60 s, 60 < WBs ≤ 120 s, WBs > 60 s, WBs > 120 s) are presented in the manuscript to reduce the data for clear message. However, the remaining WB durations (i.e., WBs ≤ 5 s, WBs ≤ 30 s, WBs ≤ 60 s, WBs ≤ 90 s, WBs ≤ 120 s, 5 < WBs ≤ 10 s, 60 < WBs ≤ 90 s, 90 < WBs ≤ 120 s) are provided in the Supplementary Material.

### Statistical Analysis

Normality of the data (gait characteristics) was checked by plotting histograms and using the Shapiro Wilk test for each environmental condition. In addition, rain clouds and box plots were used to visually check the distribution for each group and shift in distribution among groups (PD vs. HC) for each gait characteristic. To evaluate differences between PD, HC, and the impact of environment, a mixed ANOVA was performed on the data aggregation by WB duration and their combined effect (interaction) on each gait characteristic. Based on the data distribution, student *t*-test and Mann Whitney *U*-test were used to evaluate differences between the PD and HC groups. Given the exploratory nature of this analysis, we used a *p*-value < 0.05 to guide statistical interpretation and did not make adjustments for multiple comparisons (Rothman, 1990; Perneger, 1998). This is due to the inclusion of mixed ANOVA for overall statistics and area under the receiver operating characteristics curve (AUC) analysis for each gait characteristic to investigate its discriminatory power (PD vs. HC) under different environmental conditions and aggregation by WB duration. In addition, the *p*-values are provided to assess the statistical significance of between group differences. The relationship between lab and real-world gait characteristics was also assessed with the Pearson’s correlation coefficient.

### Machine Learning Classification of Parkinson’s Disease

Three different ML models were used: support vector machine (SVM), random forest (RF), and an ensemble of these two classifiers (Figure 1D; Rehman et al., 2019a,b). The ensemble model made the decision based on soft voting (probability) (Pedregosa et al., 2011). Due to the variety of data distributions, instead of training a separate model for each WB threshold, ML models were trained by combining all WB duration data. This allowed one single model to be developed, which could cater for the distribution of the entire dataset. Training performance of the models was evaluated using a 10-fold cross-validation technique on 70% of data, and separate testing was done on each WB duration threshold by keeping 30% of the data for testing. This rigorous training and testing process was repeated 10 times based on different random seed values. Classifier performance was evaluated in terms of accuracy, F1 score, AUC, sensitivity, and specificity (Rehman et al., 2019a, 2020a,b). In addition, influential gait characteristics were also identified based on their importance in RF and recursive feature elimination (RFE) technique with SVM-linear (Rehman et al., 2019a,b). Model hyperparameters were optimized with grid search. The standard python library SciKit learn was used for ML analysis.

## Results

Demographic and clinical characteristics are summarized in Table 1. There were no significant differences between the PD and HC groups for sex, age, height, mass, and BMI. People with PD had lower cognitive scores (MoCA) and reduced Activities-Specific Balance Confidence (ABC) score compared to HC (*p* < 0.05). PD participants had an average disease duration of 26 months, the majority of which were Hoehn and Yahr stage II with an average MDS-UPDRS III score of 31.5 ± 9.8.

**TABLE 1:** Demographics and clinical measures of the Parkinson’s disease (PD) and healthy controls (HC) group.

Characteristics	HC (*n* = 52) Mean ± SD	PD (*n* = 47) Mean ± SD	*p*
M/F (*n*)	28/24	32/15	0.083
Age (years)	70.39 ± 6.88	68.36 ± 8.98	0.216
Height (m)	1.69 ± 0.08	1.70 ± 0.08	0.542
Mass (kg)	81.13 ± 15.15	80.27 ± 15.67	0.786
BMI (kg/m^2^)	28.29 ± 4.23	27.62 ± 4.62	0.455
MoCA	27.61 ± 2.39	26.28 ± 3.60	**0.037**
ABCs (0–100%)	91.02 ± 11.65	80.88 ± 16.18	**0.003**
Medication (LEDD, mg/day)		415.08 ± 212.61	
Time from Clinical Diagnosis (months)		26.42 ± 5.48	
Hoehn and Yahr (*n*)		HY I–7 (15%)	
		HY II–38 (81%)	
		HY III–2 (4%)	
MDS-UPDRS III		31.53 ± 9.79	
		(HY I–16.6 ± 4.73)	
		(HY II–33.28 ± 8.81)	
		(HY III–35.5 ± 0.71)	

*M, Males; F, Females; BMI, Body Mass Index; MoCA, Montreal Cognitive Assessment; ABCs, Activities Specific Balance Confidence scale; LEDD, Levodopa Equivalent Daily Dose; MDS-UPDRS, Movement Disorder Society Unified Parkinson’s Disease Rating Scale. Bold values mean a significant difference between PD and HC.*

### Impact of Environment and Data Aggregation on Gait Characteristics

#### Overall Statistics

**FIGURE 2:**
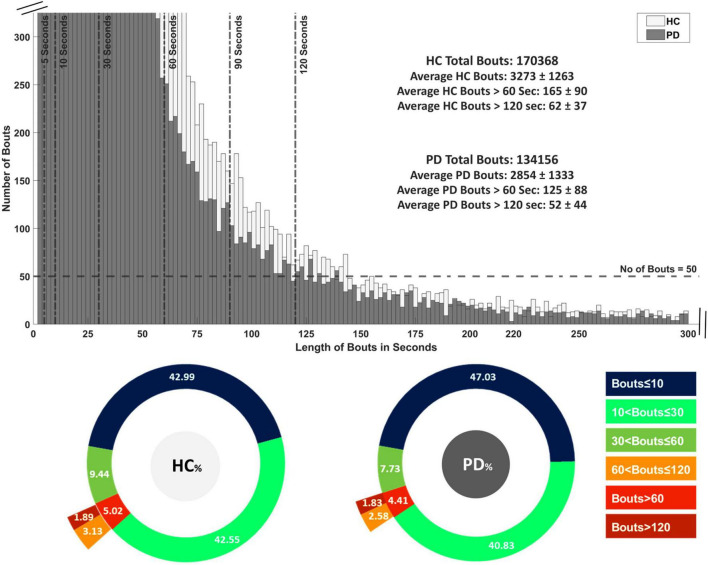
Distribution of all detected walking bouts (WBs) in Parkinson’s disease (PD) and healthy control (HC) groups in the real-world assessment of 7 days. WB are categorized intro 14 thresholds based on time in seconds followed by their average of 7 days.

**FIGURE 3:**
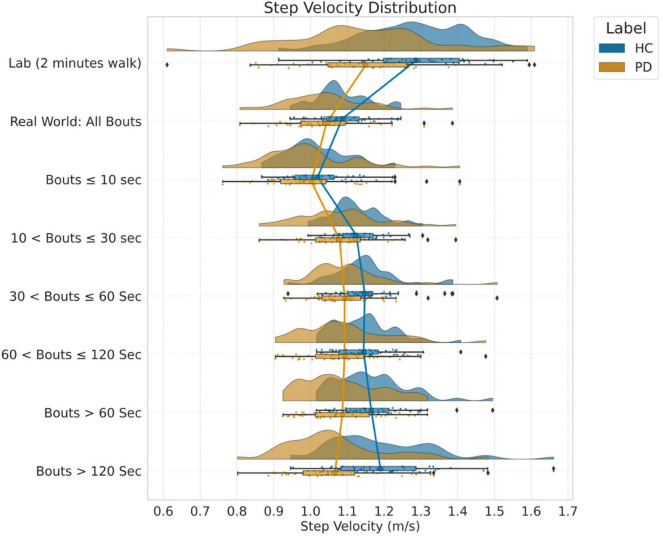
Distribution of step velocity (m/s) into different WB thresholds and average of 7 days.

**FIGURE 4:**
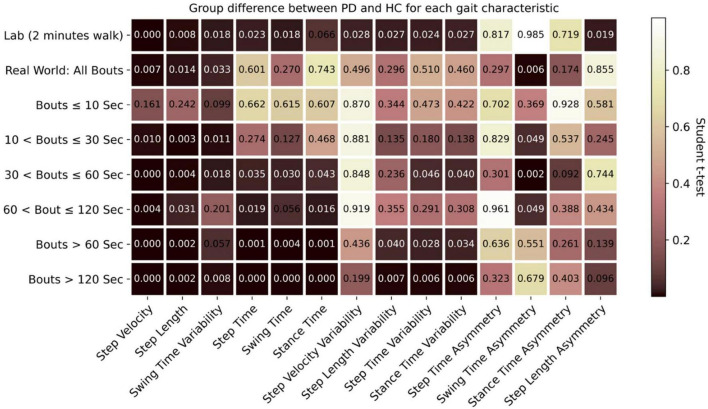
Comparison of effect of environment (lab vs. real-world) and WB duration (threshold) on discrimination between PD and HC participants (Dark highlighted color means lower *p* values).

The distribution of WB depending on duration is shown in Figure 2 and the distribution of step velocity is shown in Figure 3. Distributions for the remainder of gait characteristics are reported in Supplementary Figures 1, 2. Overall, mixed ANOVA statistical analysis results are presented in Table 2. Statistical differences between PD and HC are displayed with a heat map in Figure 4.

**TABLE 2:** Mixed ANOVA results: main effects and interactions between gait assessment environmental conditions (lab vs. real-world) and groups (PD vs. HC) for each gait characteristic.

Gait characteristics	Between participant factor: Group (HC, PD)			Within participant factor: Gait data (Lab, real-world, and all WB)			Interaction: Group × Gait		
			
	*F*	*p*	η^2^	*F*	*p*	η^2^	*F*	*p*	η^2^
Step Velocity (m/s)	9.46	0.003*	0.089	73.36	<0.001*	0.431	5.47	0.001*	0.053
Step Length (m)	9.95	0.002*	0.093	138.22	<0.001*	0.588	2.56	0.058	0.026
Swing Time Variability (s)	6.83	0.01*	0.066	158.35	<0.001*	0.62	2.25	0.085	0.23
Step Time (s)	5.45	0.022*	0.053	42.58	<0.001*	0.305	4.83	0.001*	0.047
Swing Time (s)	6.85	0.01*	0.066	66.63	<0.001*	0.407	3.67	0.007*	0.036
Stance Time (s)	4.64	0.034*	0.046	37.07	<0.001*	0.276	4.54	0.002*	0.045
Step Velocity Variability (m/s)	0.22	0.638	0.002	128.51	<0.001*	0.57	1.49	0.218	0.015
Step Length Variability (m)	1.58	0.211	0.016	58.31	<0.001*	0.375	2.11	0.097	0.021
Step Time Variability (s)	5.15	0.026*	0.5	173.13	<0.001*	0.641	2.81	0.04*	0.028
Stance Time Variability (s)	5.79	0.018*	0.056	170.86	<0.001*	0.638	3.14	0.023*	0.031
Step Time Asymmetry (s)	4.44	0.038*	0.044	1018.75	<0.001*	0.913	1.11	0.342	0.011
Swing Time Asymmetry (s)	7.79	0.006*	0.074	1117.42	<0.001*	0.92	2.64	0.047*	0.026
Stance Time Asymmetry (s)	5.05	0.027*	0.05	986.81	<0.001*	0.911	2.04	0.113	0.021
Step Length Asymmetry (m)	0.18	0.671	0.002	911.29	<0.001*	0.904	2.29	0.051	0.023

Partial eta squared (η^2^) represents effect size; F statistic (F); significance (p) indicated as *.

There was a significant interaction (Table 2) between environment (lab and real-world, including data aggregation by WB duration) and group (HC, PD) for seven characteristics (step velocity, step time, swing time, stance time, step time variability, stance time variability, and swing time asymmetry). However, the main effect of within participant factor revealed that all the gait characteristics evaluated in the lab setting were significantly different from those evaluated in the real-world setting and for all data aggregations. Similarly, the main effect of between-participant factor revealed that there were significant differences between PD and HC for all the gait characteristics except step velocity variability, step length variability, and step length asymmetry.

#### Impact of Environment [Lab vs. Real-World (All Walking Bouts)] on Between-Group Differences (Parkinson’s Disease vs. Healthy Control)

##### Lab vs. Real-World (All Walking Bouts)

During the 2-min continuous walk in the lab, both groups walked faster with a shorter step time and longer step length compared to when in the real-world regardless of WB duration. Even for the longest WB (>120 s), these characteristics (step velocity and step length) were reduced compared to the 2-min (120 s) continuous walk in the lab. As an example, the distribution of step velocity for both groups and environmental conditions under different data aggregation by WB durations is shown in Figure 3. Gait variability (swing time, step time, stance time, step length, and step velocity) was reduced when measured in the lab compared to the real-world. Gait was more symmetrical when measured in the lab (step time, stance time, swing time, and step length) for both PD and HC compared to real-world gait irrespective of WB duration.

##### Parkinson’s Disease vs. Healthy Control

Both in the lab and real-world conditions, when combining all WB durations, PD participants walked slower, with slower step time and shorter step length compared to HC (Supplementary Figure 1). Step velocity and step length were significantly different (*p* < 0.05) between PD and HC in both lab and real-world conditions (Figure 4). In the real-world, PD participants had significantly lower swing time variability compared to HC (*p* = 0.033). None of the asymmetry-based gait characteristics measured in the lab were significantly different between PD and HC, except for step length asymmetry (Figure 4). Similarly, for asymmetry-based characteristics in the real-world, only swing time asymmetry, based on the combination of all WBs, was significantly higher for PD compared to HC (*p* = 0.006) (Figure 4).

#### Effect of Real-World Data Aggregation on Gait

##### Distribution of Data Aggregation by Walking Bout Durations

All PD and HC participants had WBs across all duration thresholds. The distribution of WBs are shown in Figure 2 and Supplementary Figure 3. The majority of WBs (87% for PD and 85% for HC) were of shorter duration (≤10 s), with relatively few WB per day found over 120 s (1.8% for PD and 1.9% for HC). Overall, HC had a greater number of WB (total *n* = 170,368 from 52 HC) over 7 days of continuous assessment compared to the PD group (total *n* = 134,156 from 47 PD).

##### Effect of Data Aggregation by Walking Bout Durations on Between Group Differences

For both PD and HC groups, the slowest speed was observed during very short WBs (≤10 s) compared to long WBs > 10 s (Figure 3 and Supplementary Figures 2, 4). The most significant (*p* < 0.01) group differences between PD and HC were found in longer WBs (>60 s or 120 s) as compared to shorter WBs (Figure 4). Similarly, reduced step length and shorter step time were observed in short WBs (≤10 s) as compared to long WBs. Step time was significantly slower for PD in longer WBs such as >60 s (*p* = 0.001) and >120 s (*p* < 0.001) compared to HC. Interestingly, gait variability in the longer WBs (>60 s and >120 s) resulted in significant differences between the groups for swing time variability, step length variability, step time variability, and stance time variability. All the asymmetry-based gait characteristics behaved differently in short and long WBs. For example, in the longer WBs (>120 s), asymmetry-based gait characteristics were similar or close to lab-based gait asymmetry characteristics.

To summarize, in the real world, significant group differences (PD vs. HC) were identified for WBs longer than 60 or 120 s for all gait characteristics apart from asymmetry-based gait characteristics.

#### Association Between Gait Characteristics Collected in the Lab vs. Real-World

**FIGURE 5:**
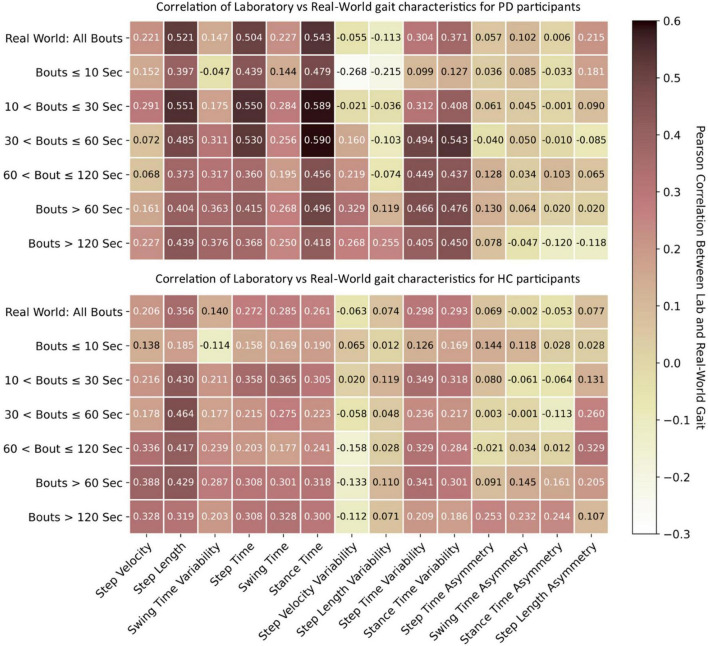
Association between lab-based gait characteristics with real-world gait. Pearson’s correlation *r* values for both PD and HC.

The association between lab and real-world gait characteristics is shown in Figure 5. In general, lab and real-world gait characteristics showed either no correlation or weak-to-moderate association with one another. However, stronger correlations were noted for the PD group compared to HC with correlations >0.5 observed for step length, step time, and stance time. Stance time resulted in the strongest correlations, followed by step time, compared to other gait characteristics, with the strongest correlation of 0.59 observed for WBs of 10–30 s and 30–60 s. Real-world gait speed resulted in a weak correlation for both PD and HC (max 0.388) with lab-based gait speed in the longer WBs > 60 or >120 s. Short WBs (≤10 s) had weak correlation compared to longer WBs (>10 s). Variability characteristics were negatively correlated between the lab and real-world gait assessments. Results for all WB durations are presented in Supplementary Figure 5.

### Impact of Environment and Data Aggregation on Classification of Parkinson’s Disease

**FIGURE 6:**
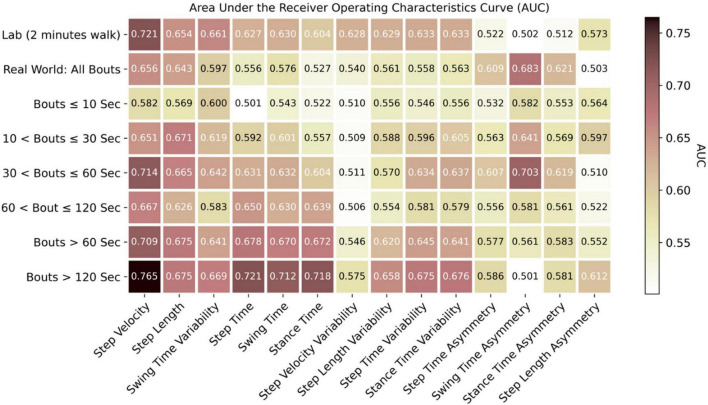
Discrimination of PD from HC based on each individual gait characteristic with area under the receiver operating characteristics curve (AUC).

**FIGURE 7:**
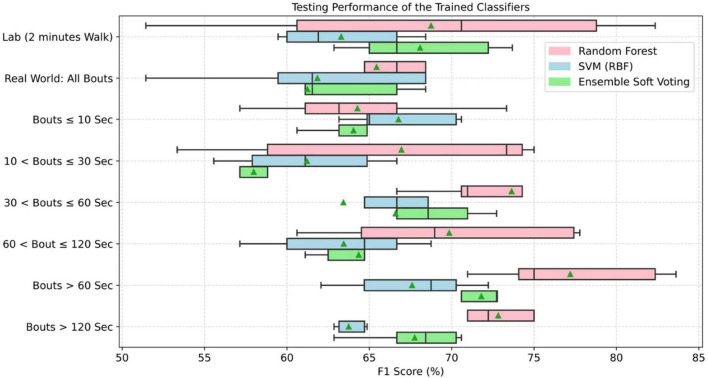
Accuracy of trained classifiers based on the lab and real-world assessment of gait grouped by WB durations (▲ means average testing accuracy across all tests on independent dataset).

Results showing the impact of environment and data aggregation by WB durations on each individual gait characteristic and ML models for classification of PD are presented in Figures 6, 7 (Supplementary Figures 6–8) and Table 3 (Supplementary Table 1).

**TABLE 3:** Evaluation metrics (accuracy, sensitivity, and specificity) of the trained classifiers on the lab and real-world test data under various walking bout (WB) durations.

Lab/WB durations	Random forest			Support vector machine			Ensemble classifier		
	Accuracy	Sensitivity	Specificity	Accuracy	Sensitivity	Specificity	Accuracy	Sensitivity	Specificity
Lab (2 min)	64.67 ± 15.02	81.62 ± 11.98	50.27 ± 19.03	51.33 ± 5.58	88.87 ± 5.56	18.12 ± 11.65	60.67 ± 5.96	88.79 ± 3.62	35.77 ± 10.71
Real-world All WB	60.67 ± 10.65	79.55 ± 15.94	43.89 ± 12.14	52.67 ± 7.23	82.95 ± 17.90	26.71 ± 5.33	53.33 ± 7.07	80.02 ± 18.85	30.72 ± 7.91
WB < 10 s	58.67 ± 9.60	78.09 ± 9.99	41.99 ± 17.87	58.67 ± 6.06	87.94 ± 9.20	32.91 ± 11.84	56.00 ± 2.79	83.75 ± 12.04	31.72 ± 7.58
10 < WB ≤ 30 s	64.67 ± 10.44	76.59 ± 15.01	54.30 ± 10.25	52.67 ± 5.96	79.41 ± 9.52	29.04 ± 6.68	52.00 ± 6.06	71.30 ± 14.78	35.43 ± 1.48
30 < WB ≤ 60 s	**72.67**±**7.96**	**80.55**±**11.19**	**65.35**±**12.46**	56.67 ± 11.30	79.98 ± 15.36	36.23 ± 12.96	62.00 ± 10.95	79.69 ± 11.79	46.37 ± 16.47
60 < WB ≤ 120 s	68.00 ± 8.03	78.97 ± 10.76	58.06 ± 8.63	60.00 ± 6.24	73.87 ± 10.63	47.88 ± 8.92	60.67 ± 5.96	75.22 ± 8.75	47.81 ± 9.31
WB > 60 s	**76.27**±**4.68**	**84.06**±**6.36**	**70.78**±**10.45**	**64.13**±**2.56**	**79.85**±**12.07**	**50.37**±**9.98**	**68.93**±**2.09**	**83.51**±**6.46**	**56.03**±**6.24**
WB > 120 s	**70.67**±**2.79**	**83.62**±**9.25**	**59.90**±**10.51**	56.00 ± 2.79	82.19 ± 7.92	32.99 ± 8.49	62.00 ± 3.80	84.87 ± 8.15	41.78 ± 8.04

*Bold values mean higher accuracy for PD classification.*

#### Machine Learning Performance in the Lab vs. Real-World

Based on the AUC for each gait characteristic discriminating PD from HC (Figure 6), real-world gait characteristics combining all WBs had relatively low AUC values compared to gait assessed in lab settings. However, the asymmetry-based characteristics in lab had lower AUC (0.51–0.57) as compared to real-world asymmetry-based gait characteristics (AUC = 0.61–0.68).

For the classification of PD, various gait characteristics were statistically significant between the groups (PD vs. HC) in lab and real-world settings. Therefore, the classifiers were trained on the overall 14 gait characteristics. During the training phase, performance of the classifiers was based on the 10-fold cross-validation and ranged between 72 and 95% (Supplementary Figure 7). These trained classifiers were tested separately on the 30% average lab and real-world test data. Random forest performed better under both environment conditions. When combining all WBs, real-world gait gave lower classification performance (accuracy: 60.67 ± 10.65) compared to lab-based gait (accuracy: 64.67 ± 15.02). This lower performance from real-world data (all WBs combined) was observed (Figure 7 for F1 score) in all the three models (random forest, support vector machine, and ensemble model). However, only RF performance was statistically significant from SVM under both environmental conditions.

#### Impact of Data Aggregation by Walking Bout Duration on Machine Learning Performance

Real-world gait characteristics had higher AUC compared to lab-based gait assessment for selected WB durations (Figure 6). The maximum AUC of 0.765 was observed for step velocity in real-world gait assessment from longer WBs (>120 s), followed by the lab-based step velocity (AUC of 0.721), 30 < WBs ≤ 60 s (AUC of 0.714), and WBs > 60 s (AUC of 0.709). All the rhythm-based gait characteristics (step time, swing time, and stance time) had an AUC around 0.7 when aggregated across longer WBs > 60 s or > 120 s. A maximum AUC of 0.703 was found in 30 < WBs ≤ 60 s for swing time asymmetry. To summarize, the maximum AUC from real-world gait characteristics were found in the longer WBs (30 < WBs ≤ 60 s, WBs > 60 s, and WBs > 120 s).

Data distribution for different thresholds of WB duration varies. Therefore, classifiers were trained on the combined data to accommodate all distributions. In addition, because there were differences in gait characteristics (discriminating PD vs. HC) for different thresholds of WB duration, classifiers were trained on all 14 gait characteristics. During the training phase, the performance of the classifier based on the 10-fold cross-validation ranged between 72 and 95% (Supplementary Figure 7). Overall, RF performed better on the new data set (30%) used for testing as compared to other classifiers (Figure 7). In addition, RF classification performance was significantly different from SVM in all WB durations except WBs < 10 s, while RF was only significantly different from ensemble model in 10 < WBs < 30 s and WBs > 120 s. Longer WBs > 60 s gave a better classification performance in discriminating PD from HC as compared to other WB durations in the real-world data. However, the classifier performance varies with different data aggregation by WB durations, which indicates that comparing gait performance from the same participants in different environments (and WB durations) can influence the classifiers. Maximum testing performance of the classifiers were obtained from 30 < WBs ≤ 60 s, WBs > 60 s, and WBs > 120 s (Table 3).

Influential gait characteristics were similar to those characteristics with a higher AUC (e.g., step velocity, step length, step time, swing time, stance time, and swing time variability; Supplementary Figure 9). Based on the importance of characteristics in the RF classifier, swing time, step velocity, stance time, swing time variability, and step length were identified as the top five characteristics. Similarly, based on the RFE with support vector machine, step velocity, step length, stance time, step time, and swing time were identified. Based on the common characteristics in the top five, step velocity, step length, swing time, and stance time were identified by both classifiers.

## Discussion

This is the first study to comprehensively investigate the impact of environment and data aggregation by WB duration on ML performance for the classification of PD. Based on the results, environment and aggregation of real-world data by WB duration influenced each individual gait characteristics for both groups and subsequent performance of ML models. We found a weak to moderate association between lab and real-world gait for both PD and HC. Based on the AUC of each gait characteristic compared to the lab, real-world gait better discriminated PD from HC, with step velocity in longer WBs (>120 s) providing the highest AUC of 0.765. In terms of PD classification, ML performance using real-world data gave better results compared to lab-based gait assessment for selected WB durations (WBs > 60 s; 30 < WB ≤ 60 s; > 120 s). Our findings show that testing environment and data aggregation (by WB duration) influence accuracy of ML performance and, therefore, classification of PD.

### Impact of Environment and Data Aggregation on Gait Characteristics

#### Lab vs. Real-World

In the present study, gait assessed in the lab appeared to give different values and results compared to the real-world gait assessed for 7 consecutive days across all gait domains (pace, rhythm, variability, asymmetry, and postural control). These findings align with previous work (Del Din et al., 2016a), where PD and HC gait was assessed in the lab (10 m walk in a straight line) and in the real world for over 7 days. A major factor explaining the differences observed between environments is that in the lab, gait is measured under controlled settings during scripted tests (reflecting capacity), whilst real-world gait is characterized by natural walking behavior executed under variable settings and conditions (reflecting performance) (Del Din et al., 2016a,b, 2019; Shah et al., 2020c). People tend to walk faster with longer steps, lower variability, and higher asymmetry in the lab compared to real-world (Del Din et al., 2016a), which is evident from the present findings and in agreement with others (Takayanagi et al., 2019). Findings from this study support previous work showing that real-world gait is more variable than lab-based gait (Del Din et al., 2016a,b, 2019).

We found that the association (correlation) between lab-based and real-world gait characteristics was weak to moderate, irrespective of WB duration, suggesting real-world and lab-based gait are measuring different aspects and constructs (i.e., performance vs. capacity) of walking (Maetzler et al., 2020). These results concur with previous work showing that walking speed during a 4 m walk had low correlation with real-world gait (Van Ancum et al., 2019). One reason for the low correlation could be the heterogeneous distribution of real-world characteristics. Moreover, other gait activities, such as turning, were not accounted for. For example, people with PD tend to turn, on average, >60 times every hour (El-Gohary et al., 2014) rather than walk in perfectly straight lines in the real-world (Galperin et al., 2019; Hillel et al., 2019), which cannot be evaluated using a tri-axial accelerometer alone.

There are many other factors influencing the complexity of real-world gait. Real-world gait is intrinsically dual task and is cognitively demanding due to complex and challenging environments in comparison to scripted gait lab tests when attention is heightened (Robles-García et al., 2015; Del Din et al., 2016a). Another important factor, especially in PD, is that medication affects gait (Ghoraani et al., 2019; Evers et al., 2020) and this is difficult to account for in the real-world where medication regimes and intake may be unknown and will impact on gait and motor fluctuations. In the present study, the lab-based gait assessment was performed one hour after medication intake in the practically defined “on” state. Therefore, we may expect to see an individual’s optimal capacity. Conversely, in the real-world, walking may take place at all points of the medication cycle, resulting in on-off fluctuations in motor function and, consequently, in gait (Ghoraani et al., 2019). This can act as a confounding factor when averaging gait characteristics across different WB durations and identifies an important area for future work to understand the effect of medication on real-world gait.

#### Parkinson’s Disease vs. Healthy Control

Gait characteristics extracted from the 2-min walk in the lab were statistically different for PD and HC compared to the real-world when combining all WBs together, except for asymmetry characteristics. These findings are difficult to compare with previous work where different protocols for gait assessment in the lab have been utilized [e.g., 10 m (Del Din et al., 2016a) or 7 m walk (Shah et al., 2020c)]. In real-world conditions, pace characteristics, such as step velocity and step length, were significantly different between PD and HC across all WBs > 10 s. Other gait characteristics (variability and asymmetry) behaved differently depending on WB duration, with differences in gait between PD and HC present for medium-to-long WB, but not for shorter WB. One possible reason for these discrepancies could be related to the algorithm, i.e., the performance of gait and step detection algorithms in shorter WBs may be challenged by noisy signals and presence of shuffling and weight transfer activities (Del Din et al., 2016a,c; Atrsaei et al., 2021). The other possible reason could be methodological: the choice of WB duration across which results are averaged may impact on gait differences, i.e., between-group gait differences found in medium WBs (which represent a high percentage of the total number of WBs) may drive results even when data are combined with results from longer WB durations as these represent a lower percentage and may offer reduced statistical power when making group comparisons (Supplementary Figures 1, 2, 6). Moreover, asymmetry that is quantified during shorter walking bouts in the real world may be linked to necessary gait adaptations to navigate complex environments. Results from medium duration WBs (e.g., 30–60 s) were comparable to those from longer WBs (>60 or >120 s) even though the latter represented only a small percentage [1.83% (PD) and 1.89% (HC)] of the total WBs. Gait characteristics, from every domain, were significantly different between PD and HC for medium-to-long WBs (30–60 s, >60, >120 s). These results are in line with previous work where the largest differences between the PD and HC groups were found in the longer WBs (>120 s) (Del Din et al., 2016a).

### Impact of Environment and Walking Bout Duration on Parkinson’s Disease Classification

Parkinson’s disease (PD) classification was more accurate for lab-based gait assessment than the real-world when all the WBs were combined. Our findings for step velocity and step length yielded greater AUC, while previous work, which focused on other biomechanical characteristics, showed that foot strike angle resulted as the gait characteristic providing highest AUC (Shah et al., 2020a,c). This could be due to the different protocol (2-min walk vs. 7 min walk), cohort characteristics, and sample size (our study group mean MDS-UPDRS III score: 31; PD *n* = 47 while Shah’s group mean MDS-UPDRS III score: 35 with PD *n* = 29). Discrepancies between studies show how, depending on the PD cohort disease severity, stage, sample size, and different gait characteristics may lead to higher classification performances as reflecting various level of impairment and progression. In addition, novel insights from our work showed that gait was more asymmetrical in the real-world, and this domain resulted in higher AUC than lab-based asymmetry results.

Because real-world gait presents a heterogeneous distribution, combining all WBs may increase spread of the data thereby “masking” significant differences between groups (Del Din et al., 2016a,b, 2019; Shah et al., 2020a,c,b; Atrsaei et al., 2021). As indicated previously, gait assessment conditions, such as lab and real-world, directly influence gait characteristics (Del Din et al., 2016a,^2019^; Van Ancum et al., 2019; Shah et al., 2020c) with optimal walking capacity found under brief testing conditions (lab) compared to real-world performance (what people actually do during everyday activities) (Maetzler et al., 2020). ML models are directly influenced by gait features obtained under these different environments, which in turn impact classification accuracy. Therefore, combining all walking bouts obtained in the real-world can result in less optimal performance in the ML classifiers explaining our findings.

#### Data Aggregation by Walking Bout Duration

No previous study has investigated the impact of WB durations on the classification of PD using ML approaches. However, within univariate gait analysis, based on Del Din et al. (2016a), longer WBs were found to be better at discriminating PD from HC. This is in contrast to Shah et al. (2020a) where 90% of participants presented with WBs less than 53 strides. Therefore, only gait characteristics from short WBs < 12 strides (<24 steps) were found to be reliable and more sensitive when discriminating PD from HC. However, due to the small sample size in Shah et al. (2020a), the effect of longer WB was possibly dampened (e.g., WBs > 60 s long can have more than 53 strides) and had not been comprehensively investigated. Other factors, such as the algorithms, sensor location, and the protocol used and experimental set-up all influence the findings. In the present study, the sensor was attached on the lower back, and for Shah et al. (2020), sensors were attached to the ankles. The comprehensive approach taken in this study (i.e., quantifying various WB durations with reasonable sample size), highlighted that longer WBs were better for discriminating PD from HC.

Overall, the random forest classifier gave better classification performance as compared to SVM (Rehman et al., 2019a). ML models gave optimal performance from WBs > 60 s followed by 30 < WBs ≤ 60 s and WBs > 120 s compared to lab and short WBs (<30 s). As discussed, real-world walking leads to various WB durations with a variety of gait speeds (Del Din et al., 2016a,^2019^). In real-world conditions, both PD and HC groups performed a large number of very short WBs (e.g., <10 s) rather than prolonged WBs (e.g., >120 s). Short WBs most likely reflect habitual behaviors and moving in a constrained environment, such as a house, while longer WBs may represent walking outdoors which influence gait characteristics and ultimately the accuracy of the classifier. This is evident from the present results as shorter WBs (<10 s) demonstrated poor discriminative performance compared to longer (>60 s) WBs (accuracy of 56–59% vs. accuracy of 68–76%).

The most influential characteristics for the classification of PD were related to pace and rhythm. Particularly, step velocity, step length, swing time, stance time, swing time variability, and step time, which were identified by both random forest and SVM. The results are in line with previous work (Rehman et al., 2019a,b, 2020a) which showed that pace (step velocity and step length) are the most common and influential characteristics for not only differentiating early-stage PD in univariate fashion (e.g., *t*-test or with AUC), but also in ML classifiers. In addition, based on the AUC values, pace characteristics (e.g., step velocity, step length) gave optimal performance in both lab and real-world data. However, the best results were obtained in the real-world for longer WBs (30–60 s, >60 s, and >120 s). The results from this study are in line with the previous work (Shah et al., 2020a,c) where the effect of WB duration influenced the AUC, and real-world gait was found to be more sensitive for discrimination purposes.

### Key Insights With Clinical Implications

• During the 2-min continuous walk in the lab, both PD and HC groups walked faster, with quicker and longer steps, lower variability, and higher asymmetry than when in the real-world, regardless of WB duration. Lab based assessment represents gait capacity, whereas real-world data reflect gait performance.
• Group differences (PD vs. HC) in gait, both in the lab and real-world conditions when combining all WBs, showed that PD participants walked slower, with shorter steps than HC. However, in the real world, significant between-group differences were influenced by WB duration (i.e., identified for WB longer than 60 or 120 s for all gait characteristics apart from those related to asymmetry). From a clinical perspective, the assessment in the clinic and outside the clinic can contain similar information. However, walking performance assessed over longer walks can offer increased sensitivity.
• Lab and real-world gait assessments assess different aspects of gait. No correlation or weak-to-moderate association was observed between the assessments. In routine clinical practice, these two streams of information can reflect different gait constructs and therefore provide complementary information to support clinical decision making.
• Individual gait characteristics measured in the real-world and averaged across all WBs (univariate analysis) had relatively low AUC values compared to gait assessed in the lab. However, specific real-world WB durations (i.e., longer 30–60 s, >60 s, >120 s) give higher AUC compared to lab-based gait assessment. This reinforces the need to consider the impact of real-world data aggregation levels for targeting specific clinical questions/aspects (e.g., classification of PD).
• With ML-based multivariate analysis, choice of environment (lab vs. real-world) and data aggregation by WB durations clearly impacted on the ML classifier performance. Our findings suggest that ML-based models should be tested on the real-world longer WBs in clinical practice as an informed pre-screening decision-making tool for PD.
• Gait assessed with wearables in the real-world paired with ML gave reasonably accurate classification performance at early stages compared to current gold standard PD clinical diagnostics. This inexpensive and objective solution motivates its adoption in clinical practice and could be a promising addition to the current clinical diagnostic toolkit and complement clinical decision-making. An accurate early diagnosis of PD is important to ensure that timely and targeted treatments (both pharmacological and non-pharmacological) can be provided.

## Limitations and Recommendations for Future Work

There are limitations to this work. From the lab-based gait assessment, only 2 min of continuous walking were utilized and compared to 60 < WBs ≤ 120 s or 90 < WBs ≤ 120 s (Supplementary Figures 1, 2) of real-world gait data. However, real-world walking comprises additional complexity, with varying visual stimuli (i.e., day, night), cognitive load (single and dual task), and motor demand (i.e., uphill, downhill), which is not reflected in lab-based gait assessments. The context (e.g., indoor vs. outdoor walking) in which short and long walking bouts happen is not measured in this work. Future work is required to develop methods for characterizing contextual information. Understanding under what scenarios gait assessment could improve the classification results. In the lab, PD participants were assessed one hour after medication intake. However, in the real-world gait assessment, we could not objectively control and assess the effect of medication on gait performance. Future studies should investigate this and the effect that medication “ON” and “OFF” states could have on the results. The ML-based findings from this early PD cohort with an average disease duration of 26 months may not be generalizable to advanced PD stages. In this work, only 14 clinically relevant gait characteristics based on the heel strike and toe-off gait events were considered. In future studies, other signal-based characteristics independent of foot contact detection should be compared. Furthermore, in routine clinical practice, misdiagnosis of PD can delay subsequent intervention and treatments. Therefore, future work should look at ML classification of PD vs. atypical parkinsonian disorders (i.e., Multiple System Atrophy and Progressive Supranuclear Palsy) to identify discriminatory gait features.

## Conclusion

In this study, we investigated the impact of environment and data aggregation by WB duration on gait characteristics and on the performance of ML models for the classification of PD. Real-world gait characteristics aggregated over medium to long WBs (e.g., WBs > 30 s) gave better discrimination performance (0.51 ≤ AUC ≤ 0.77) compared to lab-based gait characteristics (0.51 ≤ AUC ≤ 0.72), with real-world step velocity showing the highest AUC (0.77). Gait data aggregation by WB durations influenced ML classification performance. ML models applied to real-world gait showed better classification performance compared to lab data. Overall, RF trained on 14 gait characteristics aggregated over WBs > 60 s gave better performance (F1 score = 77.20 ± 5.51%) as compared to lab-based data. Findings from this study suggest that choice of environment and data aggregation by WB duration are important to achieve maximum discrimination performance and have a direct impact on ML performance for PD classification.

## Data Availability Statement

All the relevant data is either reported in the form of table or displayed in figure. The plots of all the digital gait characteristics are presented in the Supplementary Material. Due to data privacy and sharing agreement, raw dataset is not publically available. However, it can be made available upon reasonable request from the corresponding author. Requests to access these datasets should be directed to SD, silvia.deldin@ncl.ac.uk.

## Ethics Statement

The studies involving human participants were reviewed and approved by the Newcastle and North Tyneside Research Ethics Committee (REC No. 09/H0906/82). Study procedures were conducted according to Declaration of Helsinki. The patients/participants provided their written informed consent to participate in this study.

## Author Contributions

RR conceptualized and designed the study, and performed data analysis, statistical analysis, drafting, and critical revision of the manuscript. SD helped in conceptualization of this study, interpretation of data, and critical revision of the manuscript for important intellectual content. YG and JS provided support for statistical analysis, interpretation, and critical revision of the manuscript for important intellectual content. LA, AY, and LR were involved in interpretation of data and critical revision of the manuscript. All authors contributed to the article and approved the submitted version.

## Author Disclaimer

Content in this publication reflects the authors’ view and neither IMI nor the European Union, EFPIA, or any Associated Partners are responsible for any use that may be made of the information contained herein.

## Conflict of Interest

The authors declare that the research was conducted in the absence of any commercial or financial relationships that could be construed as a potential conflict of interest.

## Publisher’s Note

All claims expressed in this article are solely those of the authors and do not necessarily represent those of their affiliated organizations, or those of the publisher, the editors and the reviewers. Any product that may be evaluated in this article, or claim that may be made by its manufacturer, is not guaranteed or endorsed by the publisher.
