# Supplementary material for: Investigating the Impact of Environment and Data Aggregation by Walking Bout Duration on Parkinson’s Disease Classification Using Machine Learning
Source: Front Aging Neurosci. 2022 Mar 22;14:808518. doi: 10.3389/fnagi.2022.808518 (PMC8981298; doi:10.3389/fnagi.2022.808518)
Supplement: Supplementary file 1 [file Data_Sheet_1.PDF]

Supplementary Material

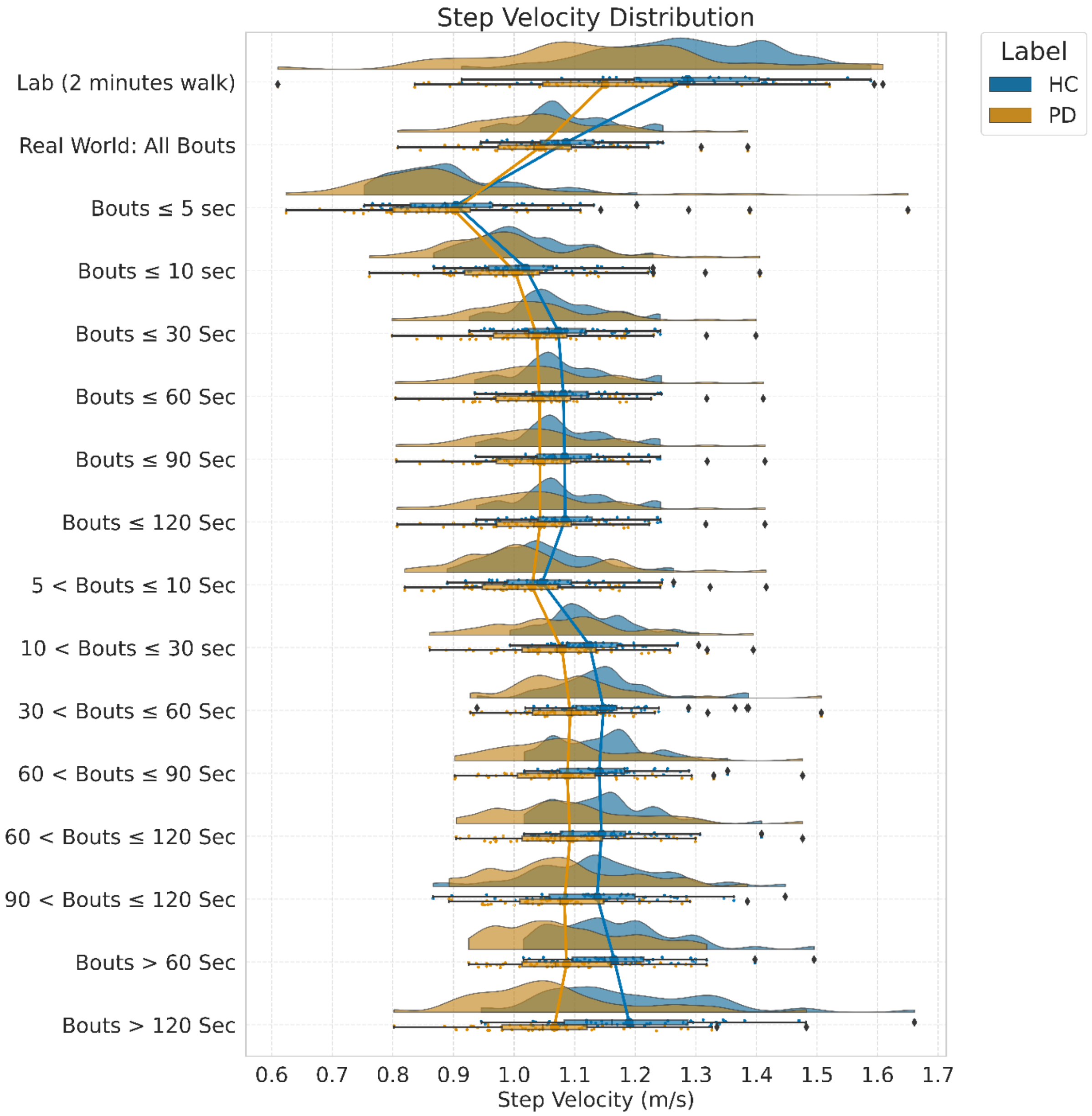

Supplementary Figure 1. Distribution of step velocity (m/s) into different WB thresholds followed by average of 7 days

(A)

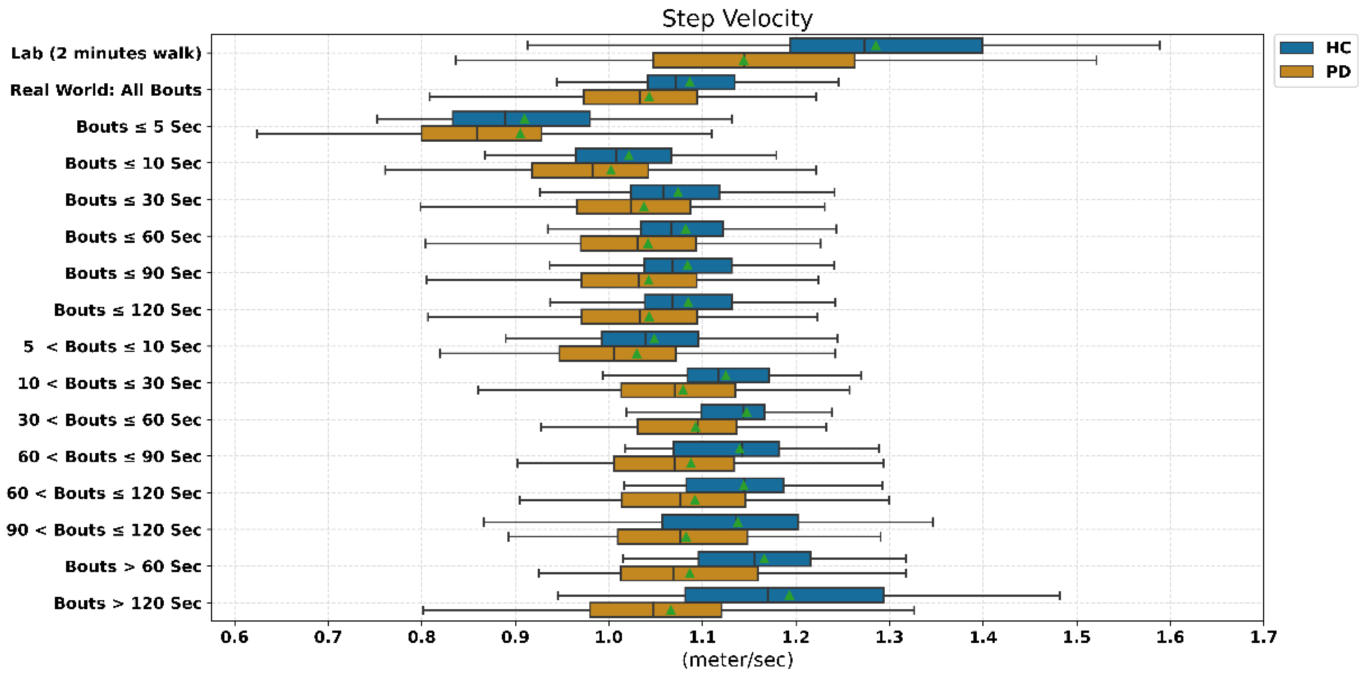
Step Velocity
Lab (2 minutes walk)
Real World: All Bouts
Bouts ≤ 5 Sec
Bouts ≤ 10 Sec
Bouts ≤ 30 Sec
Bouts ≤ 60 Sec
Bouts ≤ 90 Sec
Bouts ≤ 120 Sec
5 < Bouts ≤ 10 Sec
10 < Bouts ≤ 30 Sec
30 < Bouts ≤ 60 Sec
60 < Bouts ≤ 90 Sec
60 < Bouts ≤ 120 Sec
90 < Bouts ≤ 120 Sec
Bouts > 60 Sec
Bouts > 120 Sec
HC
PD
0.6 0.7 0.8 0.9 1.0 1.1 1.2 1.3 1.4 1.5 1.6 1.7
(meter/sec)

(B)

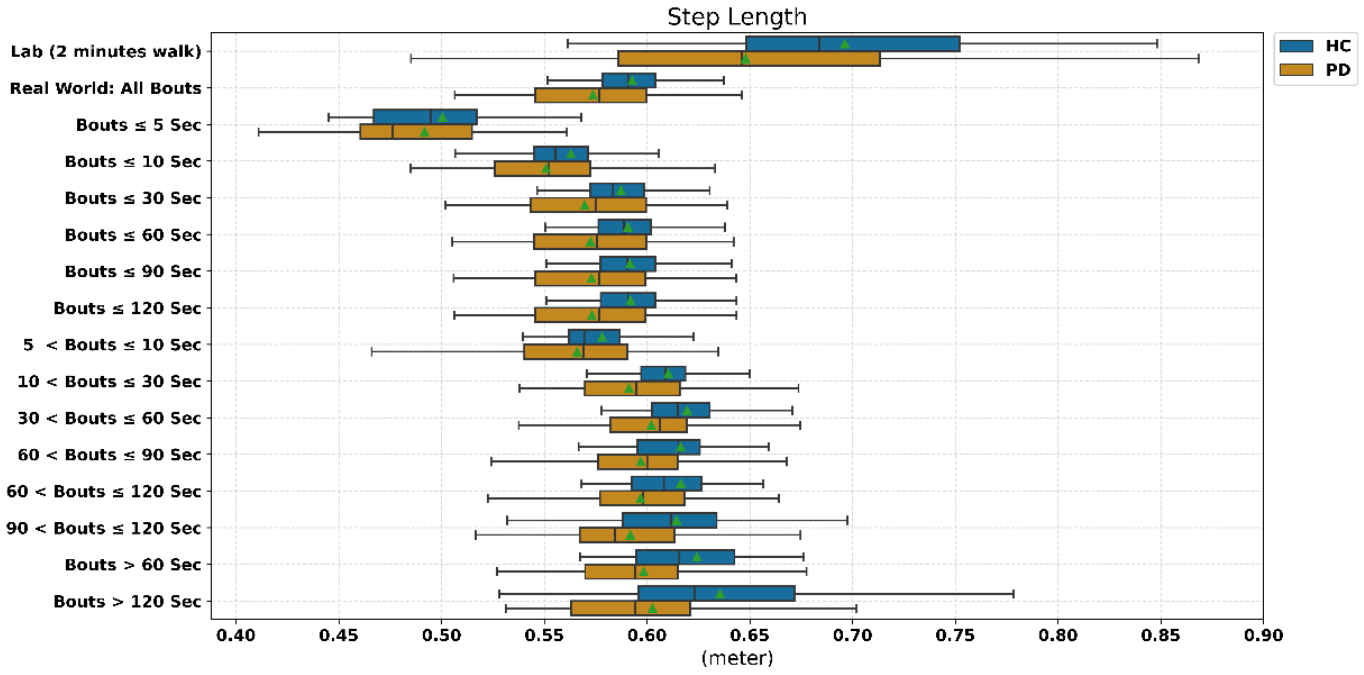
Step Length
Lab (2 minutes walk)
Real World: All Bouts
Bouts ≤ 5 Sec
Bouts ≤ 10 Sec
Bouts ≤ 30 Sec
Bouts ≤ 60 Sec
Bouts ≤ 90 Sec
Bouts ≤ 120 Sec
5 < Bouts ≤ 10 Sec
10 < Bouts ≤ 30 Sec
30 < Bouts ≤ 60 Sec
60 < Bouts ≤ 90 Sec
60 < Bouts ≤ 120 Sec
90 < Bouts ≤ 120 Sec
Bouts > 60 Sec
Bouts > 120 Sec
HC
PD
0.40 0.45 0.50 0.55 0.60 0.65 0.70 0.75 0.80 0.85 0.90
(meter)

(C)

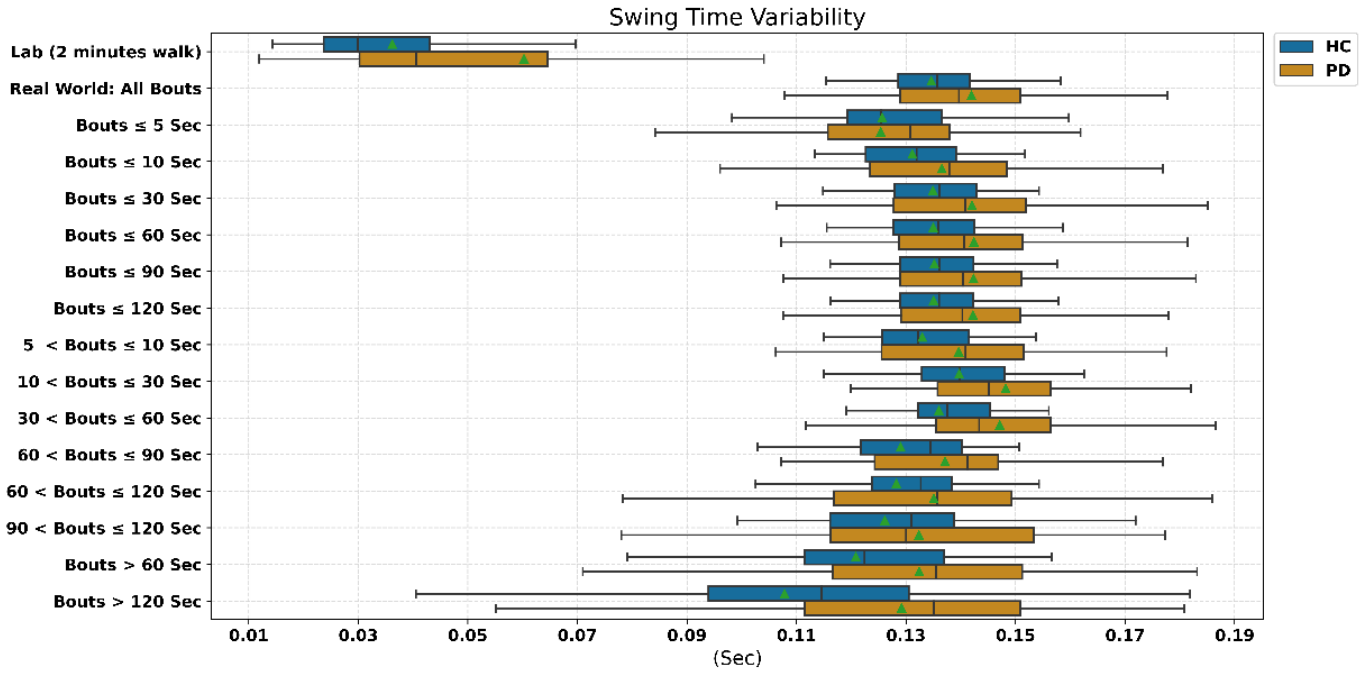
Swing Time Variability
HC
PD
Lab (2 minutes walk)
Real World: All Bouts
Bouts ≤ 5 Sec
Bouts ≤ 10 Sec
Bouts ≤ 30 Sec
Bouts ≤ 60 Sec
Bouts ≤ 90 Sec
Bouts ≤ 120 Sec
5 < Bouts ≤ 10 Sec
10 < Bouts ≤ 30 Sec
30 < Bouts ≤ 60 Sec
60 < Bouts ≤ 90 Sec
60 < Bouts ≤ 120 Sec
90 < Bouts ≤ 120 Sec
Bouts > 60 Sec
Bouts > 120 Sec
0.01
0.03
0.05
0.07
0.09
0.11
0.13
0.15
0.17
0.19
(Sec)

(D)

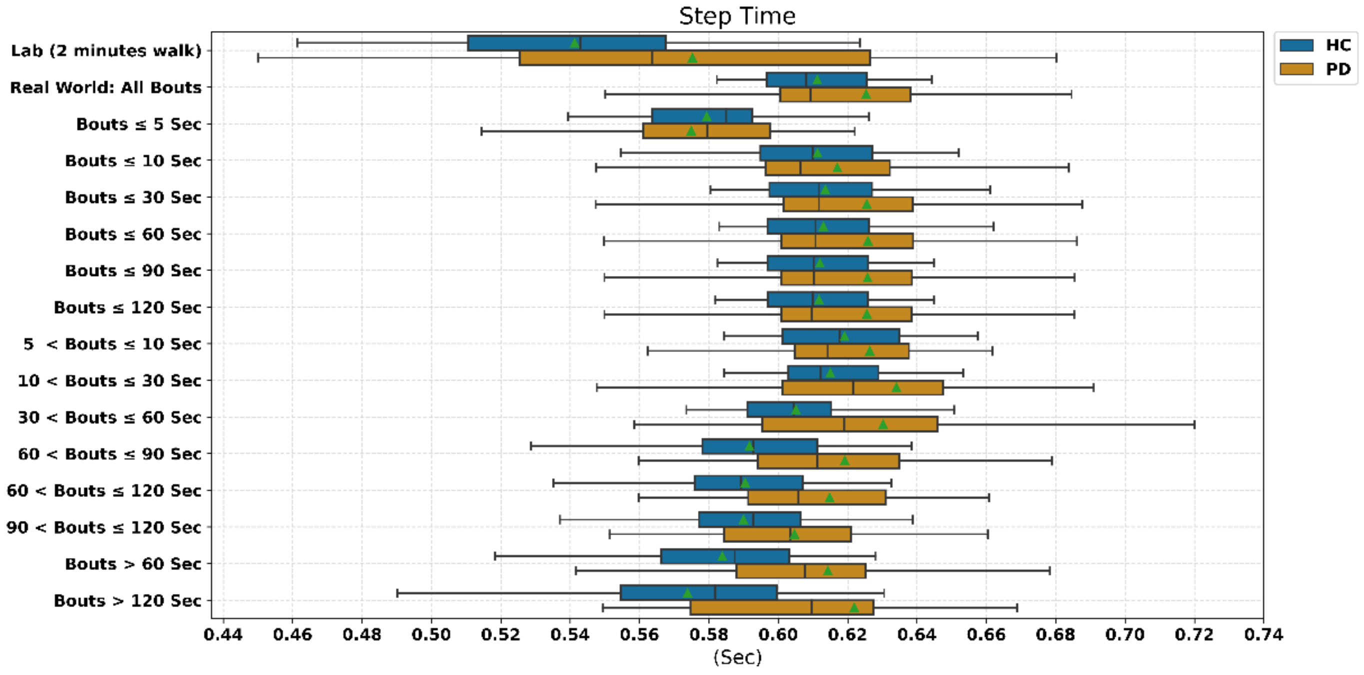
Step Time
HC
PD
Lab (2 minutes walk)
Real World: All Bouts
Bouts ≤ 5 Sec
Bouts ≤ 10 Sec
Bouts ≤ 30 Sec
Bouts ≤ 60 Sec
Bouts ≤ 90 Sec
Bouts ≤ 120 Sec
5 < Bouts ≤ 10 Sec
10 < Bouts ≤ 30 Sec
30 < Bouts ≤ 60 Sec
60 < Bouts ≤ 90 Sec
60 < Bouts ≤ 120 Sec
90 < Bouts ≤ 120 Sec
Bouts > 60 Sec
Bouts > 120 Sec
0.44
0.46
0.48
0.50
0.52
0.54
0.56
0.58
0.60
0.62
0.64
0.66
0.68
0.70
0.72
0.74
(Sec)

(E)

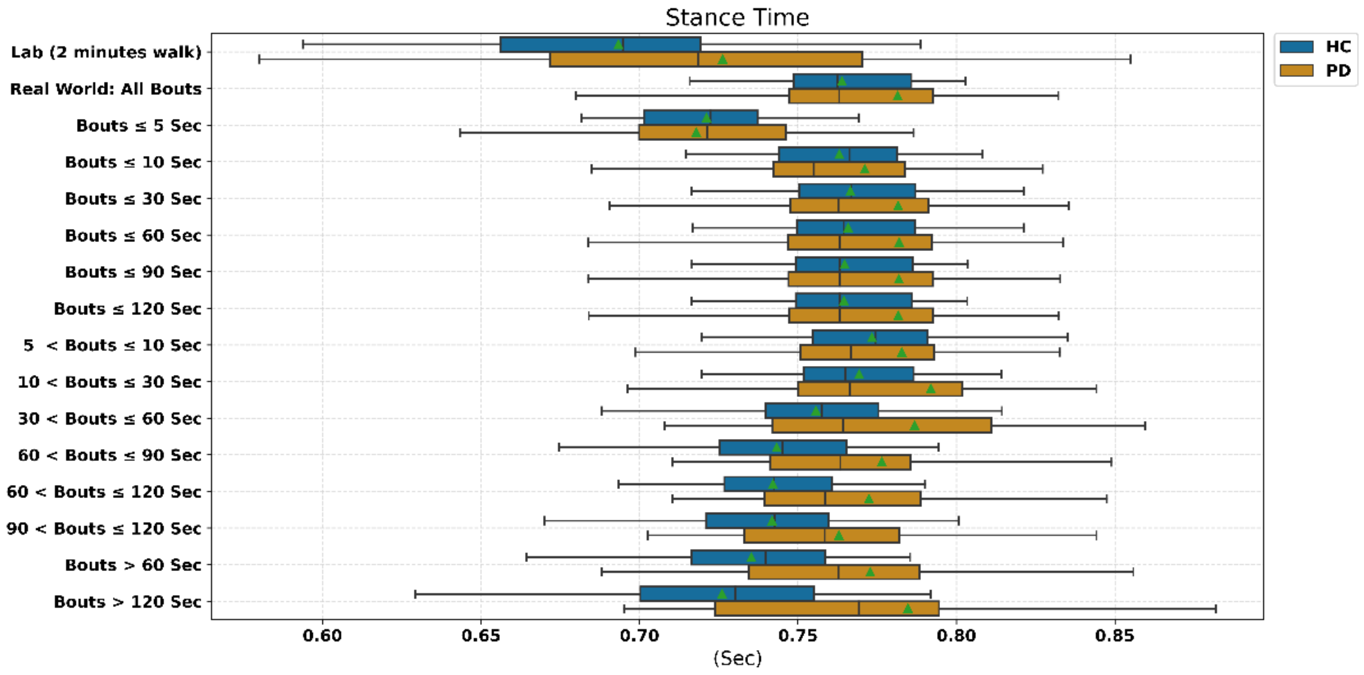

Stance Time
Lab (2 minutes walk)
Real World: All Bouts
Bouts ≤ 5 Sec
Bouts ≤ 10 Sec
Bouts ≤ 30 Sec
Bouts ≤ 60 Sec
Bouts ≤ 90 Sec
Bouts ≤ 120 Sec
5 < Bouts ≤ 10 Sec
10 < Bouts ≤ 30 Sec
30 < Bouts ≤ 60 Sec
60 < Bouts ≤ 90 Sec
60 < Bouts ≤ 120 Sec
90 < Bouts ≤ 120 Sec
Bouts > 60 Sec
Bouts > 120 Sec
0.60
0.65
0.70
0.75
0.80
0.85
(Sec)
HC
PD

(F)

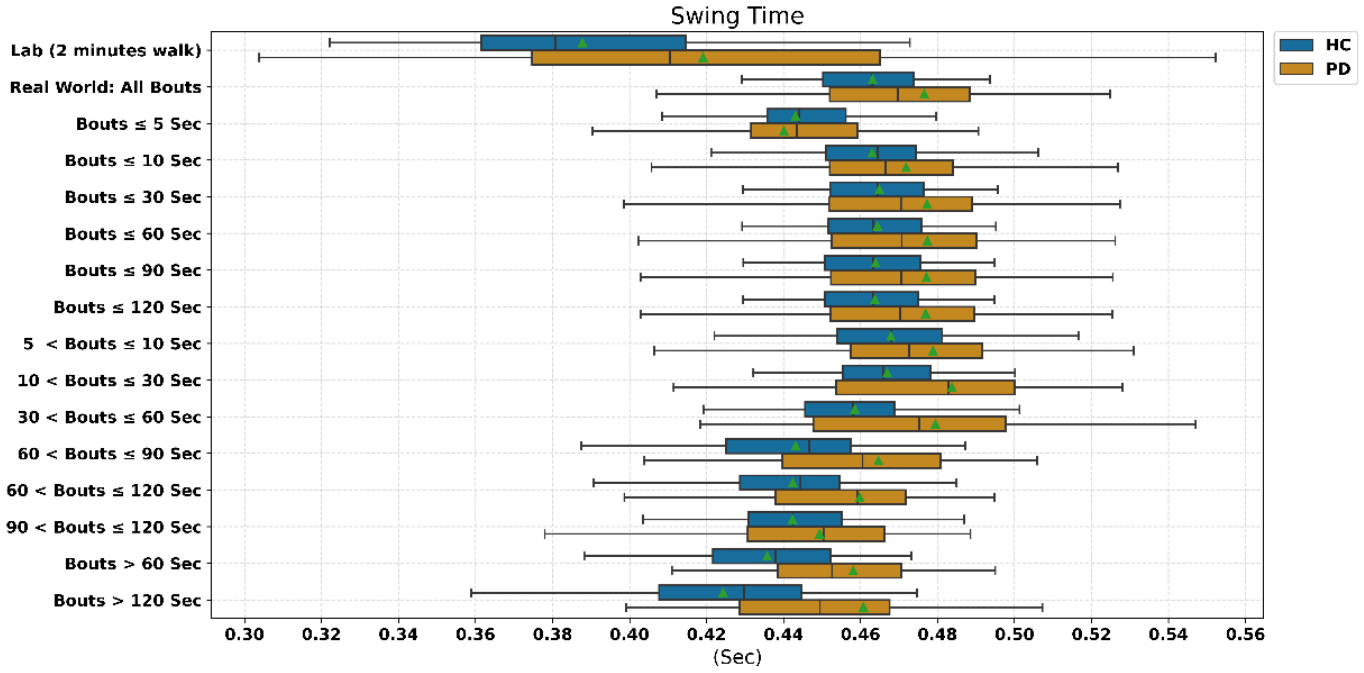

Swing Time
Lab (2 minutes walk)
Real World: All Bouts
Bouts ≤ 5 Sec
Bouts ≤ 10 Sec
Bouts ≤ 30 Sec
Bouts ≤ 60 Sec
Bouts ≤ 90 Sec
Bouts ≤ 120 Sec
5 < Bouts ≤ 10 Sec
10 < Bouts ≤ 30 Sec
30 < Bouts ≤ 60 Sec
60 < Bouts ≤ 90 Sec
60 < Bouts ≤ 120 Sec
90 < Bouts ≤ 120 Sec
Bouts > 60 Sec
Bouts > 120 Sec
0.30
0.32
0.34
0.36
0.38
0.40
0.42
0.44
0.46
0.48
0.50
0.52
0.54
0.56
(Sec)
HC
PD

(G)

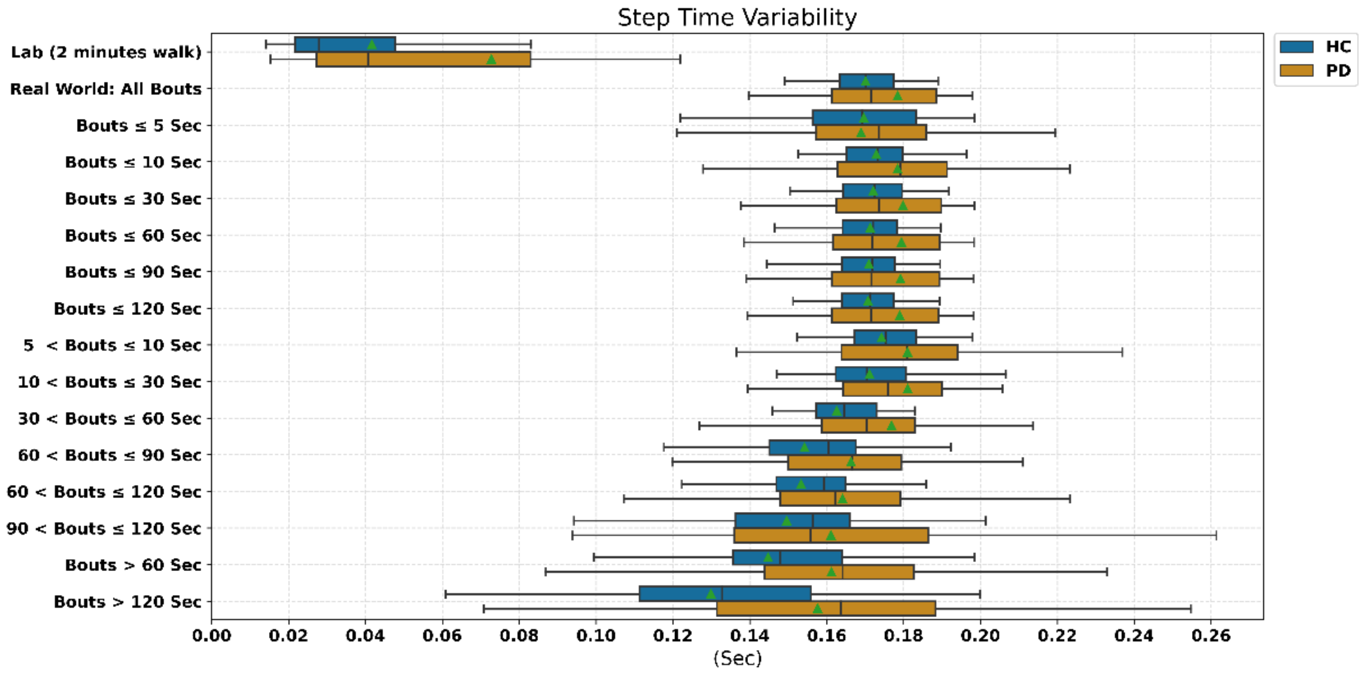
Step Time Variability
HC
PD
Lab (2 minutes walk)
Real World: All Bouts
Bouts ≤ 5 Sec
Bouts ≤ 10 Sec
Bouts ≤ 30 Sec
Bouts ≤ 60 Sec
Bouts ≤ 90 Sec
Bouts ≤ 120 Sec
5 < Bouts ≤ 10 Sec
10 < Bouts ≤ 30 Sec
30 < Bouts ≤ 60 Sec
60 < Bouts ≤ 90 Sec
60 < Bouts ≤ 120 Sec
90 < Bouts ≤ 120 Sec
Bouts > 60 Sec
Bouts > 120 Sec
0.00 0.02 0.04 0.06 0.08 0.10 0.12 0.14 0.16 0.18 0.20 0.22 0.24 0.26
(Sec)

(H)

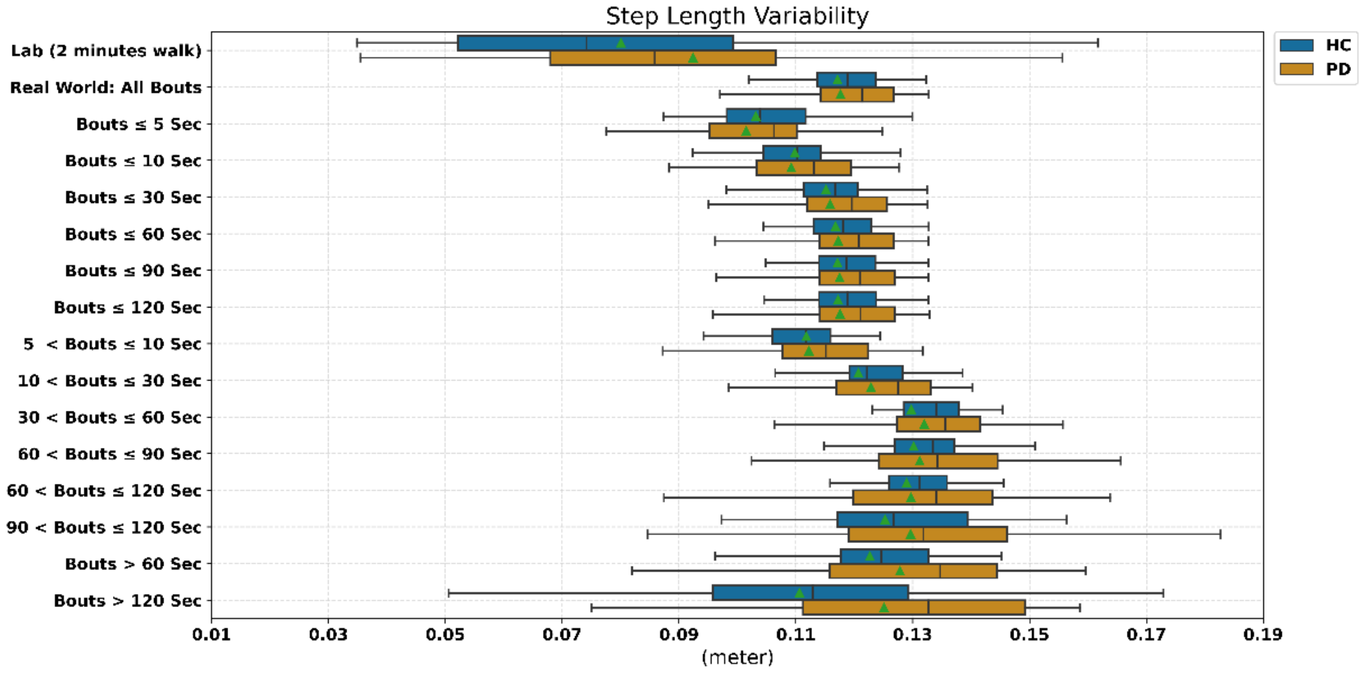
Step Length Variability
HC
PD
Lab (2 minutes walk)
Real World: All Bouts
Bouts ≤ 5 Sec
Bouts ≤ 10 Sec
Bouts ≤ 30 Sec
Bouts ≤ 60 Sec
Bouts ≤ 90 Sec
Bouts ≤ 120 Sec
5 < Bouts ≤ 10 Sec
10 < Bouts ≤ 30 Sec
30 < Bouts ≤ 60 Sec
60 < Bouts ≤ 90 Sec
60 < Bouts ≤ 120 Sec
90 < Bouts ≤ 120 Sec
Bouts > 60 Sec
Bouts > 120 Sec
0.01 0.03 0.05 0.07 0.09 0.11 0.13 0.15 0.17 0.19
(meter)

(I)

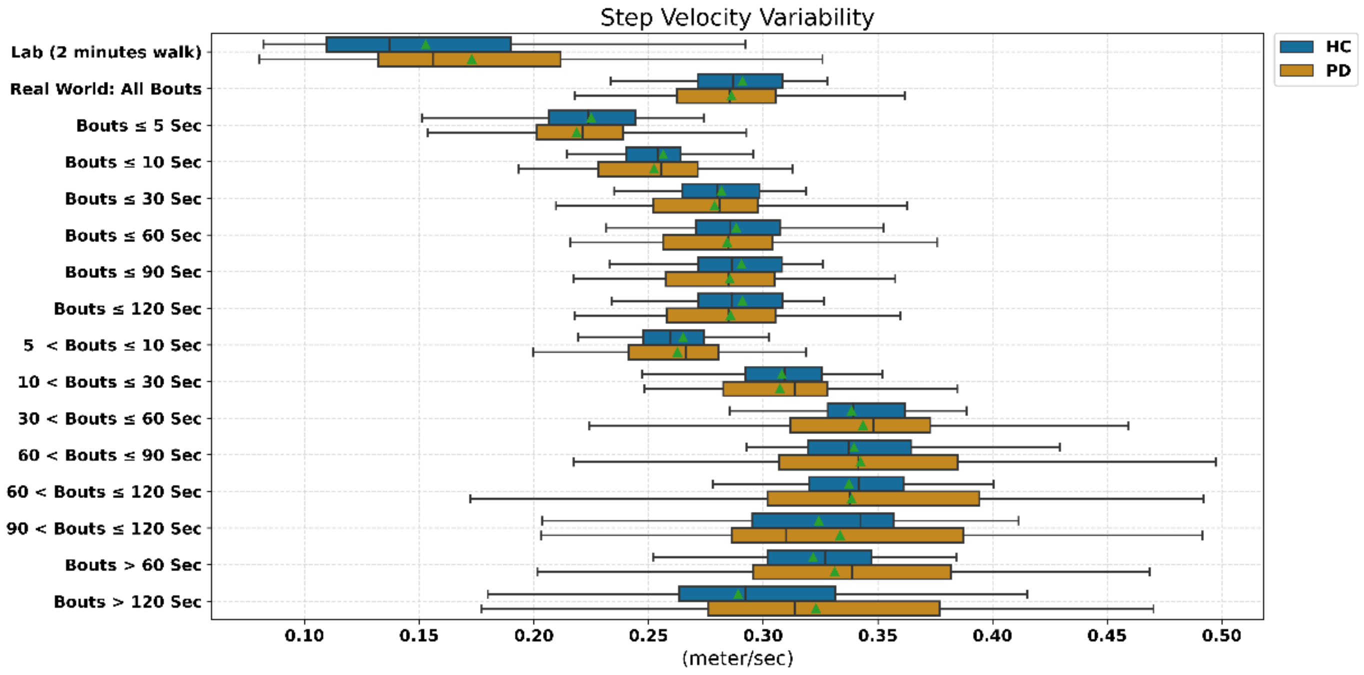

Step Velocity Variability
Lab (2 minutes walk)
Real World: All Bouts
Bouts ≤ 5 Sec
Bouts ≤ 10 Sec
Bouts ≤ 30 Sec
Bouts ≤ 60 Sec
Bouts ≤ 90 Sec
Bouts ≤ 120 Sec
5 < Bouts ≤ 10 Sec
10 < Bouts ≤ 30 Sec
30 < Bouts ≤ 60 Sec
60 < Bouts ≤ 90 Sec
60 < Bouts ≤ 120 Sec
90 < Bouts ≤ 120 Sec
Bouts > 60 Sec
Bouts > 120 Sec
0.10 0.15 0.20 0.25 0.30 0.35 0.40 0.45 0.50
(meter/sec)
HC
PD

(J)

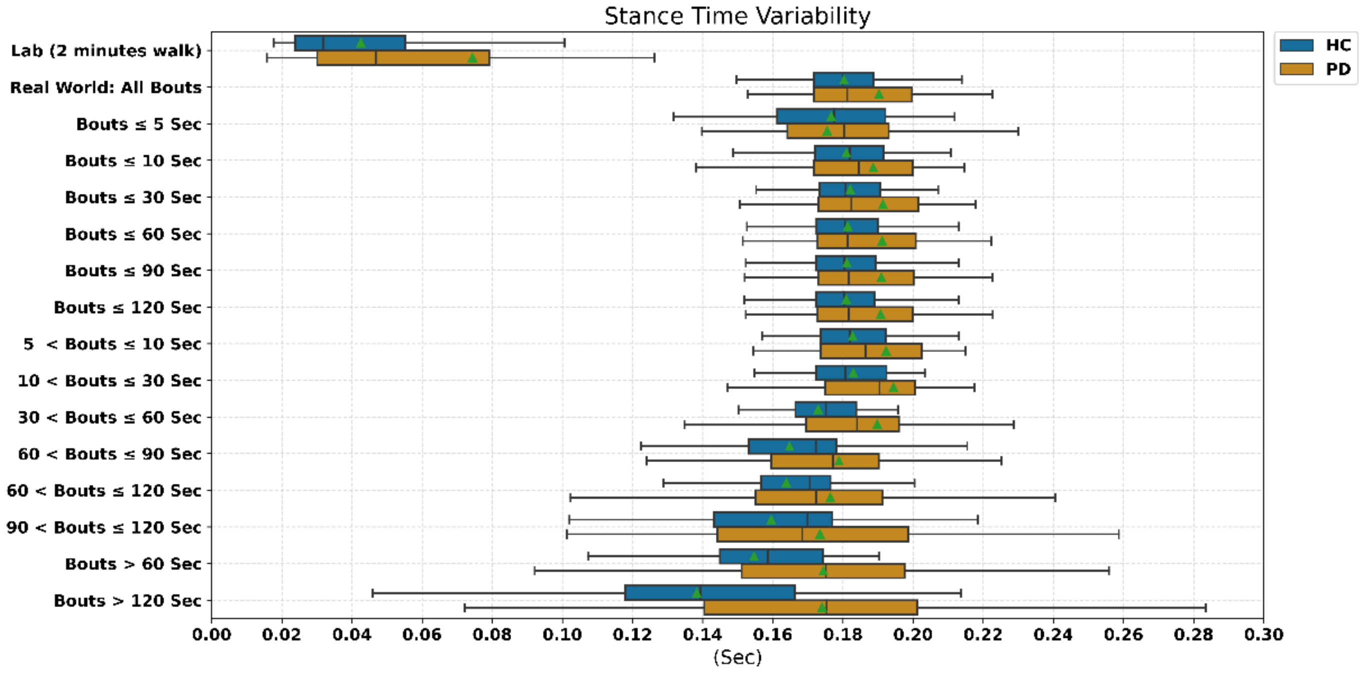

Stance Time Variability
Lab (2 minutes walk)
Real World: All Bouts
Bouts ≤ 5 Sec
Bouts ≤ 10 Sec
Bouts ≤ 30 Sec
Bouts ≤ 60 Sec
Bouts ≤ 90 Sec
Bouts ≤ 120 Sec
5 < Bouts ≤ 10 Sec
10 < Bouts ≤ 30 Sec
30 < Bouts ≤ 60 Sec
60 < Bouts ≤ 90 Sec
60 < Bouts ≤ 120 Sec
90 < Bouts ≤ 120 Sec
Bouts > 60 Sec
Bouts > 120 Sec
0.00 0.02 0.04 0.06 0.08 0.10 0.12 0.14 0.16 0.18 0.20 0.22 0.24 0.26 0.28 0.30
(Sec)
HC
PD

(K)

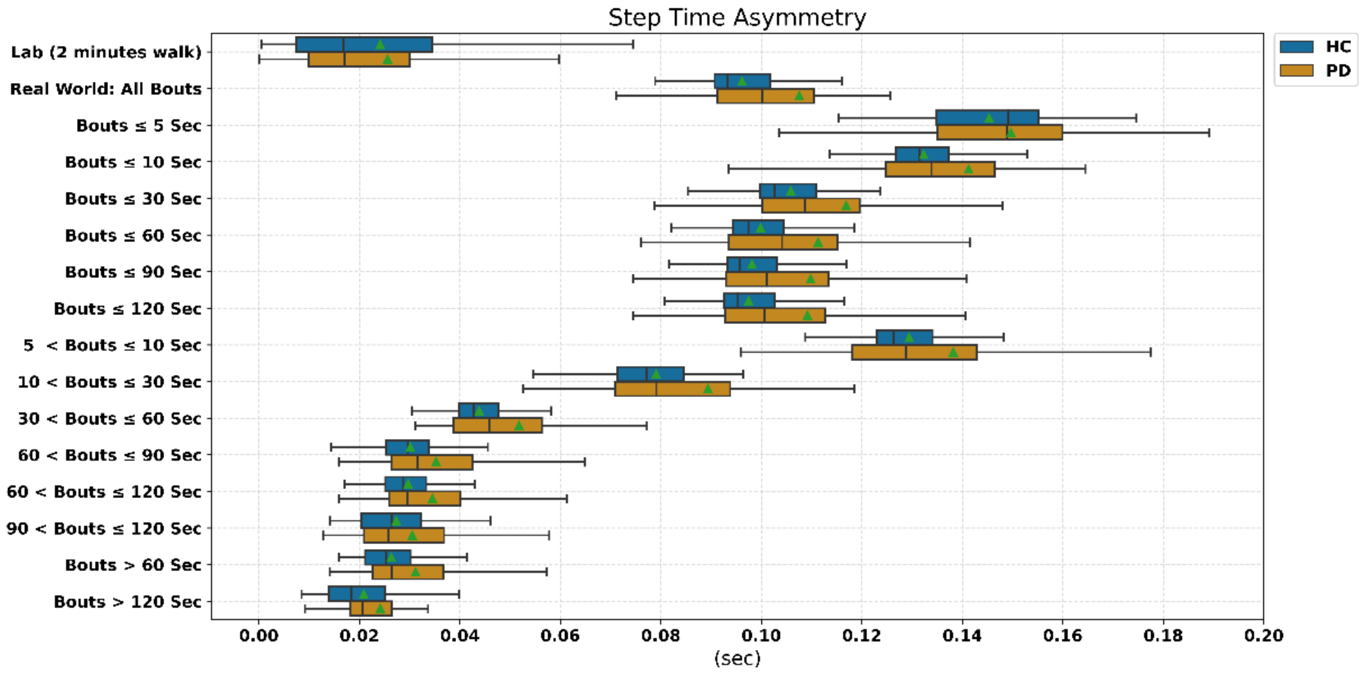
Step Time Asymmetry
HC
PD
Lab (2 minutes walk)
Real World: All Bouts
Bouts ≤ 5 Sec
Bouts ≤ 10 Sec
Bouts ≤ 30 Sec
Bouts ≤ 60 Sec
Bouts ≤ 90 Sec
Bouts ≤ 120 Sec
5 < Bouts ≤ 10 Sec
10 < Bouts ≤ 30 Sec
30 < Bouts ≤ 60 Sec
60 < Bouts ≤ 90 Sec
60 < Bouts ≤ 120 Sec
90 < Bouts ≤ 120 Sec
Bouts > 60 Sec
Bouts > 120 Sec
0.00
0.02
0.04
0.06
0.08
0.10
0.12
0.14
0.16
0.18
0.20
(sec)

(L)

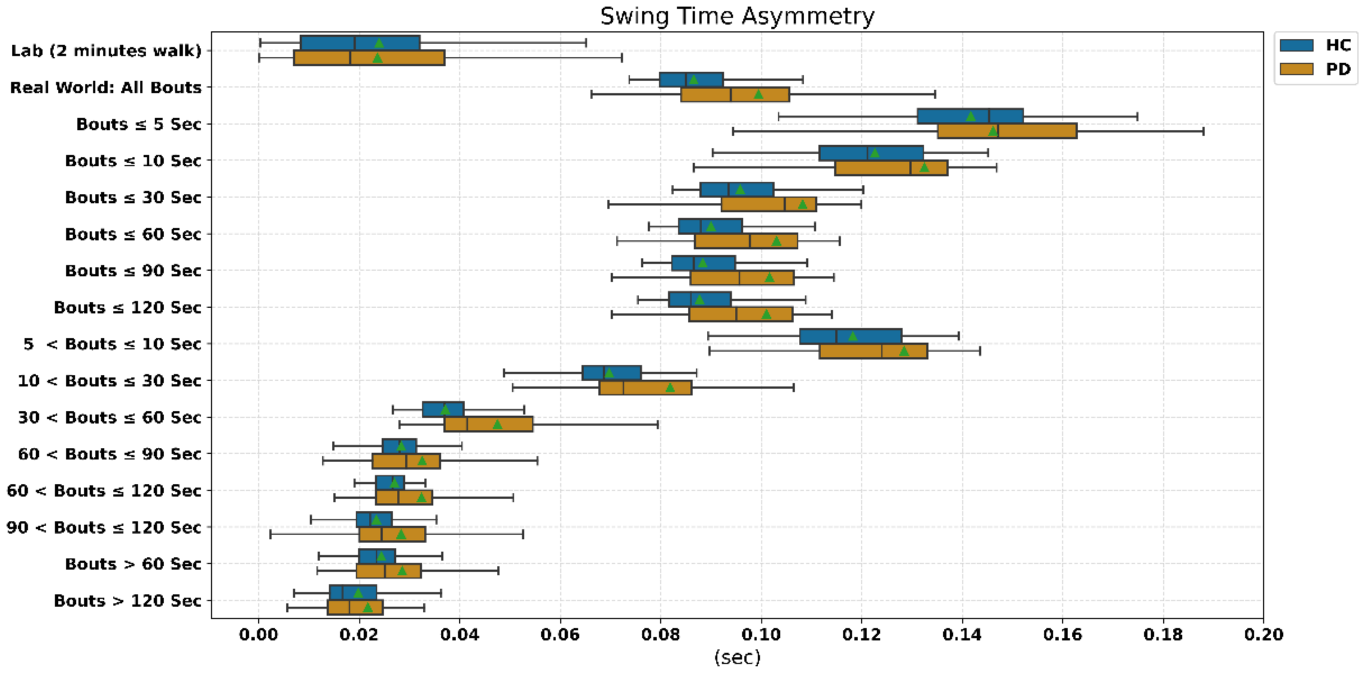
Swing Time Asymmetry
HC
PD
Lab (2 minutes walk)
Real World: All Bouts
Bouts ≤ 5 Sec
Bouts ≤ 10 Sec
Bouts ≤ 30 Sec
Bouts ≤ 60 Sec
Bouts ≤ 90 Sec
Bouts ≤ 120 Sec
5 < Bouts ≤ 10 Sec
10 < Bouts ≤ 30 Sec
30 < Bouts ≤ 60 Sec
60 < Bouts ≤ 90 Sec
60 < Bouts ≤ 120 Sec
90 < Bouts ≤ 120 Sec
Bouts > 60 Sec
Bouts > 120 Sec
0.00
0.02
0.04
0.06
0.08
0.10
0.12
0.14
0.16
0.18
0.20
(sec)

(M)

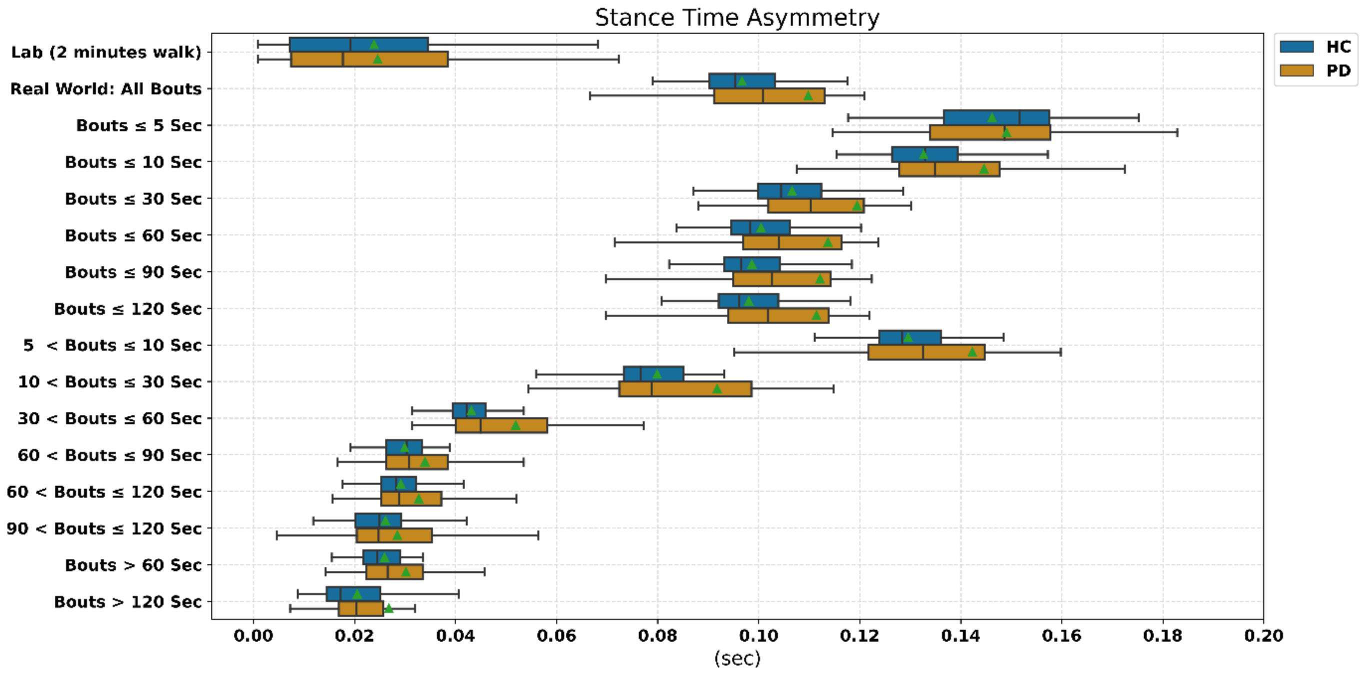

(N)

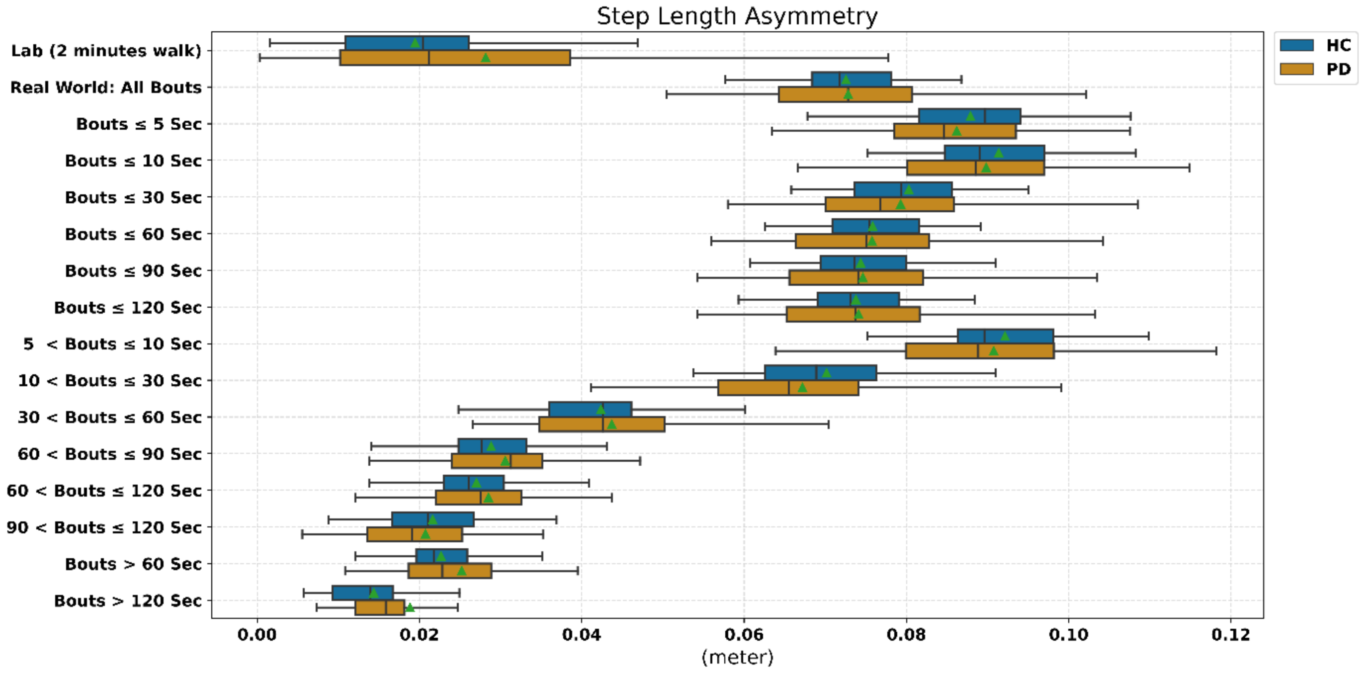

Supplementary Figure 2. Boxplots of 14 gait characteristics (A-N) under different thresholds

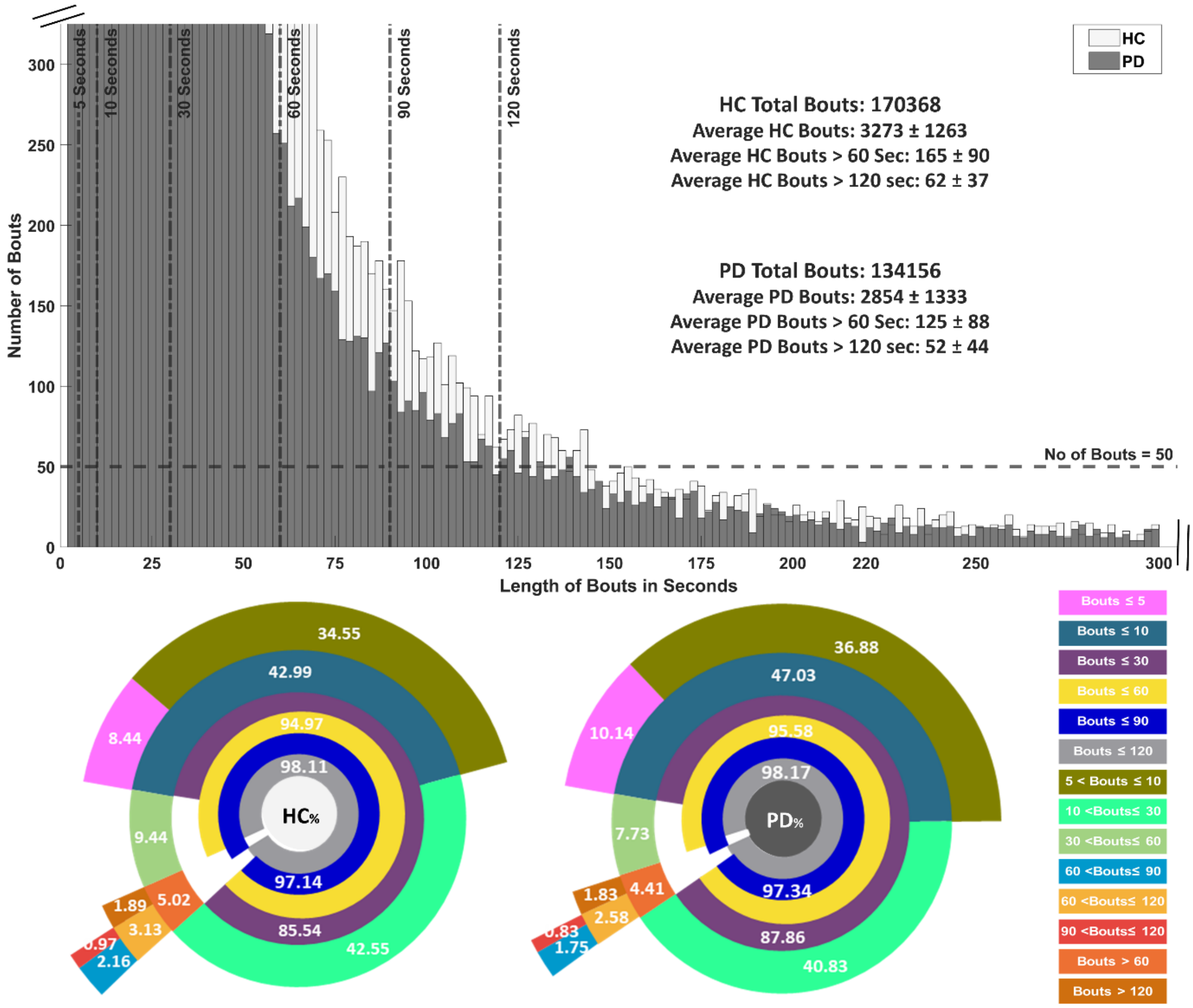

Supplementary Figure 3. Distribution of the all detected WB in PD and HC group in the real world assessment of 7 days; WB are categorised intro 14 thresholds based on time in seconds followed by their average of 7 days

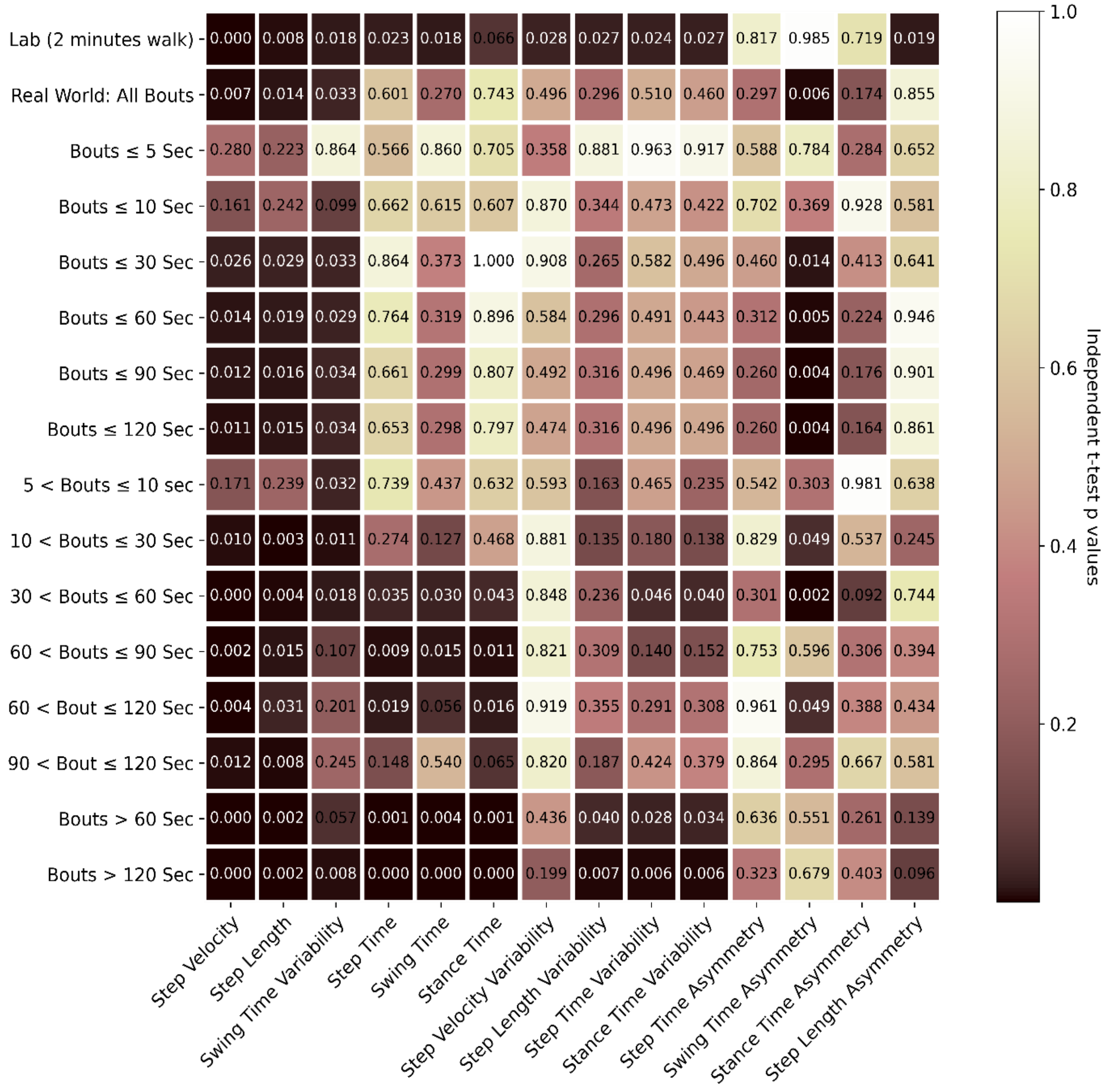

Supplementary Figure 4. Comparison of effect of environment (lab vs real-world) and bout duration (threshold) on discrimination between PD and HC participants (Dark highlighted colour means lower p values)

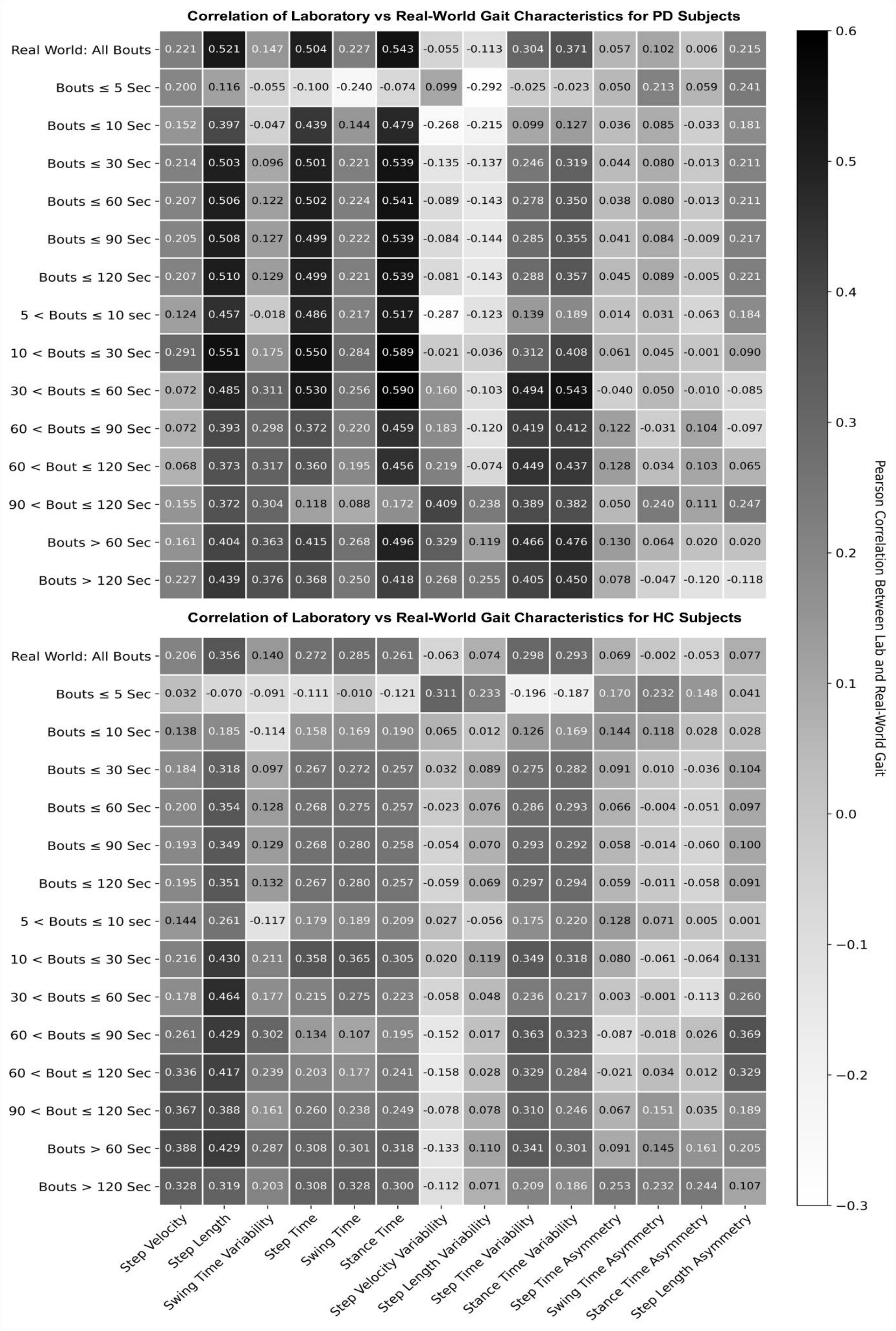

Supplementary Figure 5. Correlation of lab-based gait characteristics with real-world gait

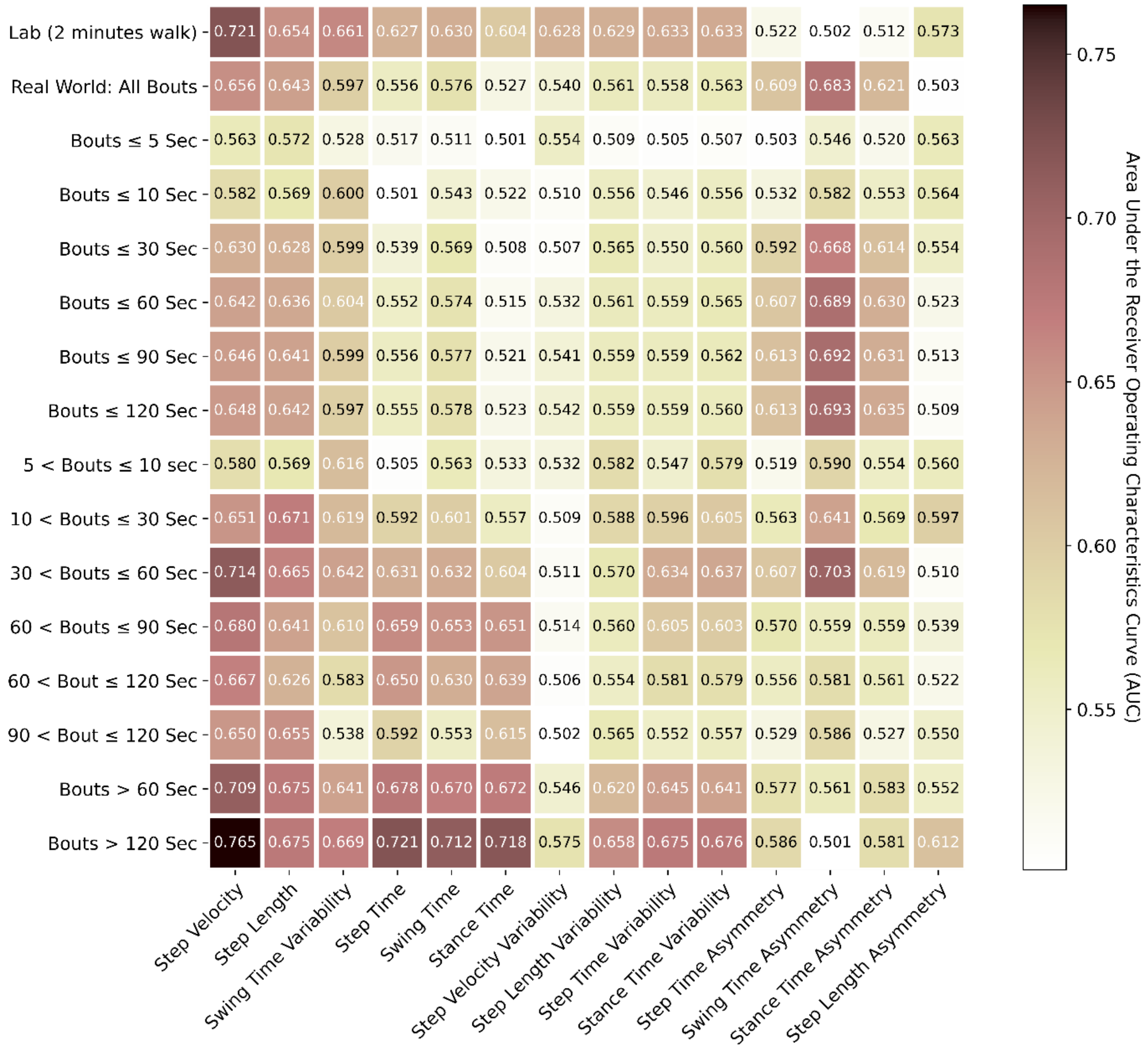

Supplementary Figure 6. Discrimination of PD from HC based on each individual gait characteristic with area under the receiver operating characteristics curve (AUC)

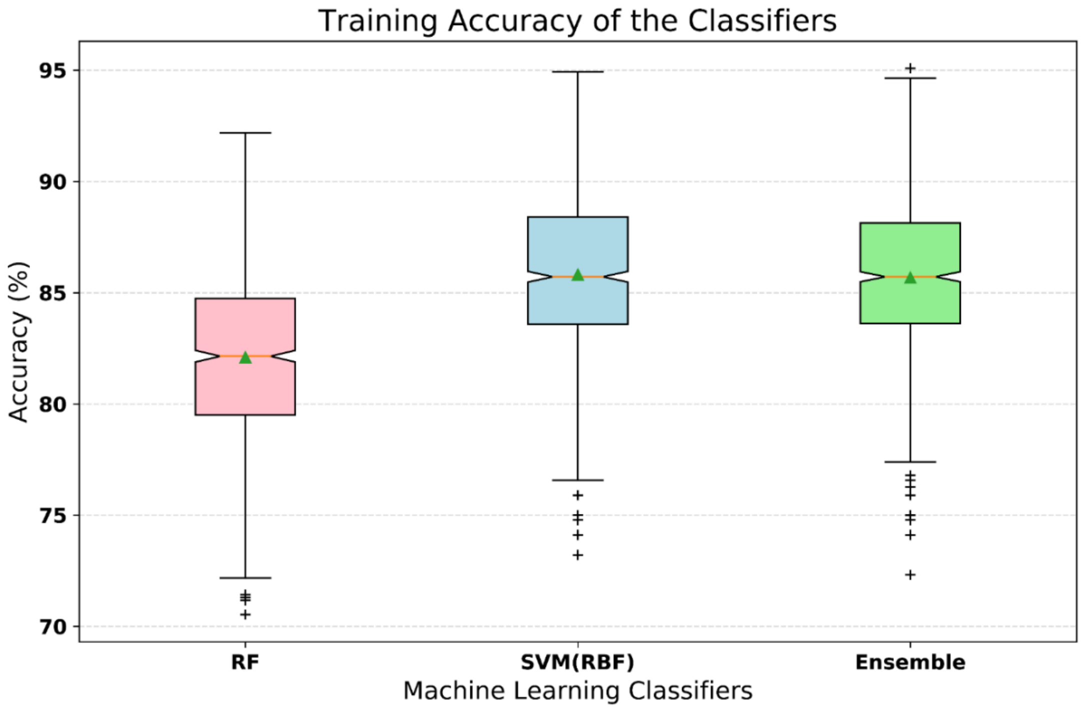

Supplementary Figure 7. Training accuracy of the classifiers based on 10-fold cross-validation by combining the distribution of the overall data

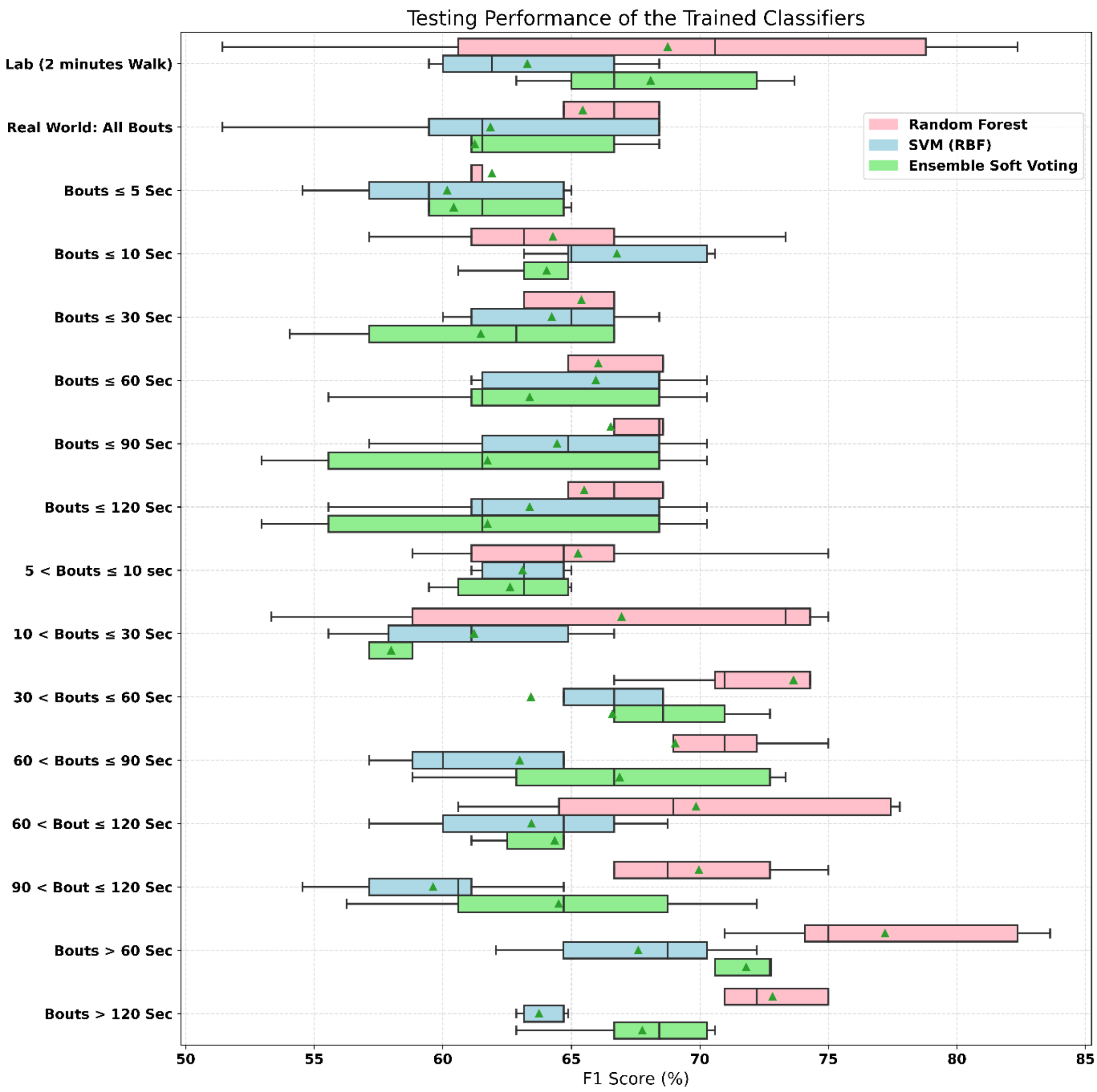

Supplementary Figure 8. Testing accuracy of the trained classifiers based on the lab and real-world assessment of gait under various bout lengths

Supplementary Table 1. Evaluation metrics of the trained classifiers on the lab and real-world test data under various bout lengths

Lab/Bout Lengths	Random Forest			Support Vector Machine			Ensemble Classifier		
	Accuracy	Sensitivity	Specificity	Accuracy	Sensitivity	Specificity	Accuracy	Sensitivity	Specificity
**Lab (2 minutes)**	64.67±15.02	81.62±11.98	50.27±19.03	51.33±5.58	88.87±5.56	18.12±11.65	60.67±5.96	88.79±3.62	35.77±10.71
**Real-world All Bouts**	60.67±10.65	79.55±15.94	43.89±12.14	52.67±7.23	82.95±17.90	26.71±5.33	53.33±7.07	80.02±18.85	30.72±7.91
**Bouts < 5 sec**	54.00±3.65	79.31±6.50	31.51±7.87	52.67±4.35	76.96±15.61	31.73±9.57	51.33±6.06	79.82±15.66	26.56±9.46
**Bouts < 10 sec**	58.67±9.60	78.09±9.99	41.99±17.87	58.67±6.06	87.94±9.20	32.91±11.84	56.00±2.79	83.75±12.04	31.72±7.58
**Bouts < 30 sec**	59.33±8.94	80.86±10.06	40.39±15.33	54.67±5.58	86.69±11.53	27.19±11.68	52.67±5.96	81.55±17.85	27.86±7.10
**Bouts < 60 sec**	60.67±10.11	81.07±13.74	42.72±13.40	57.33±5.48	88.12±11.82	30.72±7.91	54.67±6.91	84.20±15.65	29.29±7.41
**Bouts < 90 sec**	62.00±9.60	80.98±16.15	45.23±11.97	56.00±5.96	85.53±14.75	30.72±7.91	53.33±7.82	81.53±18.78	29.29±7.41
**Bouts < 120 sec**	60.67±10.90	79.73±16.37	43.89±12.14	54.67±6.91	84.20±15.65	29.29±7.41	53.33±7.82	81.53±18.78	29.29±7.41
**5 < Bouts ≤ 10 sec**	60.00±8.16	79.63±11.69	42.99±10.98	54.00±3.65	83.72±10.08	28.13±11.17	54.00±2.79	82.41±12.90	29.46±10.32
**10 < Bouts ≤ 30 sec**	64.67±10.44	76.59±15.01	54.30±10.25	52.67±5.96	79.41±9.52	29.04±6.68	52.00±6.06	71.30±14.78	35.43±1.48
**30 < Bouts ≤ 60 sec**	**72.67±7.96**	**80.55±11.19**	**65.35±12.46**	56.67±11.30	79.98±15.36	36.23±12.96	62.00±10.95	79.69±11.79	46.37±16.47
**60 < Bouts ≤ 90 sec**	68.67±8.37	73.21±9.12	64.08±16.53	58.67±7.67	74.89±10.38	44.13±8.45	63.33±8.50	77.88±7.42	50.42±11.52
**60 < Bouts ≤ 120 sec**	68.00±8.03	78.97±10.76	58.06±8.63	60.00±6.24	73.87±10.63	47.88±8.92	60.67±5.96	75.22±8.75	47.81±9.31
**90 < Bouts ≤ 120 sec**	68.67±2.98	81.38±9.38	58.80±5.20	54.00±4.35	75.64±9.89	36.88±7.43	60.67±5.96	79.97±12.36	45.27±5.48
**Bouts > 60 sec**	**76.27±4.68**	**84.06±6.36**	**70.78±10.45**	**64.13±2.56**	**79.85±12.07**	**50.37±9.98**	**68.93±2.09**	**83.51±6.46**	**56.03±6.24**
**Bouts > 120 sec**	**70.67±2.79**	**83.62±9.25**	**59.90±10.51**	56.00±2.79	82.19±7.92	32.99±8.49	62.00±3.80	84.87±8.15	41.78±8.04

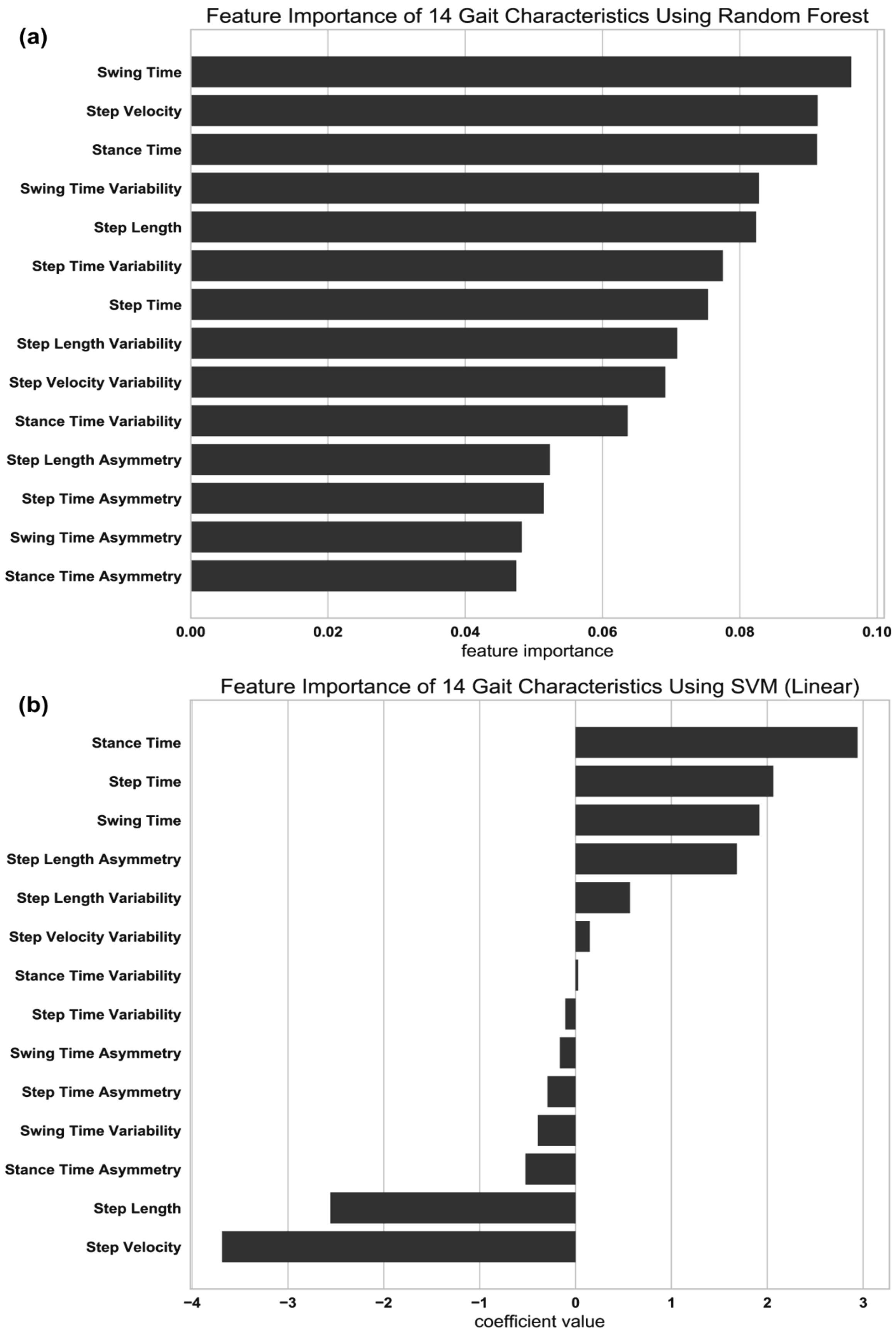

Supplementary Figure 9. Important characteristics identified during the classification of PD with (a) Random Forest Classifier, (b) Support Vector Machine Classifier (longer bar means more influential)
